# Recent Applications of Propylene Carbonate as a Solvent in Sustainable Organic Synthesis

**DOI:** 10.1002/cssc.71001

**Published:** 2026-09-01

**Authors:** Laura G. Aguilar‐Sanchez, Christopher R. Jones

**Affiliations:** ^1^ Department of Chemistry Queen Mary University of London London UK

**Keywords:** Green Chemistry, propylene carbonate, solvent substitution, sustainable organic synthesis, sustainable solvents

## Abstract

Solvents are essential components for organic synthesis and are widely used across various industries. However, conventional solvents are frequently toxic volatile organic compounds (VOCs) that contribute significantly to waste generation and environmental pollution. This has driven the need to develop sustainable alternatives that maintain efficacy without the associated hazardous properties. Propylene carbonate (PC) has emerged as a promising green alternative for sustainable organic synthesis. As a polar aprotic solvent with similar physicochemical properties to acetonitrile and acetone, PC has been shown to afford high yields and selectivities comparable to those of traditional solvents in a wide range of synthetic transformations. It possesses favourable characteristics, such as low toxicity, high dielectric constant and solvation capacity, biodegradability and low vapour pressure. High thermal stability provides significant capacity for reusability across multiple reaction cycles with minimal loss of efficiency, a crucial feature for reaction scale‐up. Moreover, PC can be readily prepared from propylene oxide and carbon dioxide, an abundant waste greenhouse gas, in a 100% atom‐economical process. With an increasing number of reports on the topic, this paper presents an updated review of both the preparation and applications of PC, addressing methodological advances and new transformations.

## Graphical abstract

This review evaluates propylene carbonate (PC) as a non‐toxic green solvent in organic synthesis, highlighting its performance in key reactions including cross‐couplings, asymmetric hydrogenations, peptide synthesis and biocatalysis. Across these processes, PC delivers yields comparable to conventional and alternative green solvents. Performance is considered relative to the physicochemical properties of PC, whilst limitations, scalability and future perspectives are also discussed.

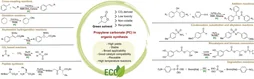

## 1 Introduction

Solvents are essential in organic synthesis as they are widely used as reaction media across various sectors, including academia and the chemical and pharmaceutical industries, where they typically account for most of the process mass. However, many conventional solvents are volatile organic compounds (VOCs) that release pollutants into the atmosphere and indirectly contribute to global warming [1]. The flammability and toxicity of many solvents can also present serious health risks, ranging from skin and eye irritations to carcinogenicity [2]. Furthermore, the amount of waste generated by solvent use has put them under increased focus with the lens of modern chemistry.

In this context, and following the principles of Green Chemistry outlined by Anastas, there is a clear need to develop sustainable alternatives to traditional organic solvents that maintain effectiveness without the associated toxic and hazardous properties [3]. Transitioning to green solvents, however, means more than just a simple swap; it requires identifying and studying media that offer comparable or better performance whilst addressing sustainability and safety concerns, waste generation and energy efficiency. Ideal green solvents should be non‐toxic, non‐volatile, biodegradable and work well with a wide range of transformations [4].

Among emerging green solvents, cyclic carbonates, particularly propylene carbonate (PC), have gained significant interest as promising candidates for sustainable organic synthesis due to their commercial availability, low production cost and favourable properties [5, 6]. PC is a polar aprotic solvent with similar characteristics to traditional organic solvents like acetonitrile (MeCN) and acetone. It is biodegradable, non‐corrosive and has a low vapour pressure, high boiling point and low toxicity. Additionally, PC is readily prepared from propylene oxide via CO_2_ valorisation; the fixation of atmospheric CO_2_ aligns with the Green Chemistry principles of 100% atom economy (principle 2) and exploitation of renewable feedstocks (principle 7).

Although the available literature supports PC as a greener solvent alternative in many organic transformations, its use alone does not necessarily guarantee the overall sustainability of a chemical process, which also depends on factors such as reagent selection, energy requirements, waste generation and process design. In other words, PC offers clear advantages, but its use must still be evaluated within the broader context of the entire synthetic workflow. The use of PC in organic synthesis was first reviewed in 2016 by Forero et al. [7]; however, since then, a range of methodological advances and new applications have been reported. As such, this review provides an updated assessment of the role of PC as a solvent in sustainable organic synthesis.

### 1.1 Polar Aprotic Solvents in Organic Synthesis

Solvents are an essential tool across many industries, particularly in the production of coatings, varnishes, adhesives, cosmetics and pharmaceutical compounds, which represent approximately 46% of global solvent use [8]. The pharmaceutical industry accounts for approximately 9% of global consumption, with most transformations performed in large volumes of solvent to control the reaction rate and product selectivity. Consequently, solvent use consistently accounts for 80%–90% of the mass utilisation in a typical pharmaceutical or chemical operation and represents over 80% of the total waste [9].

**FIGURE 1:**
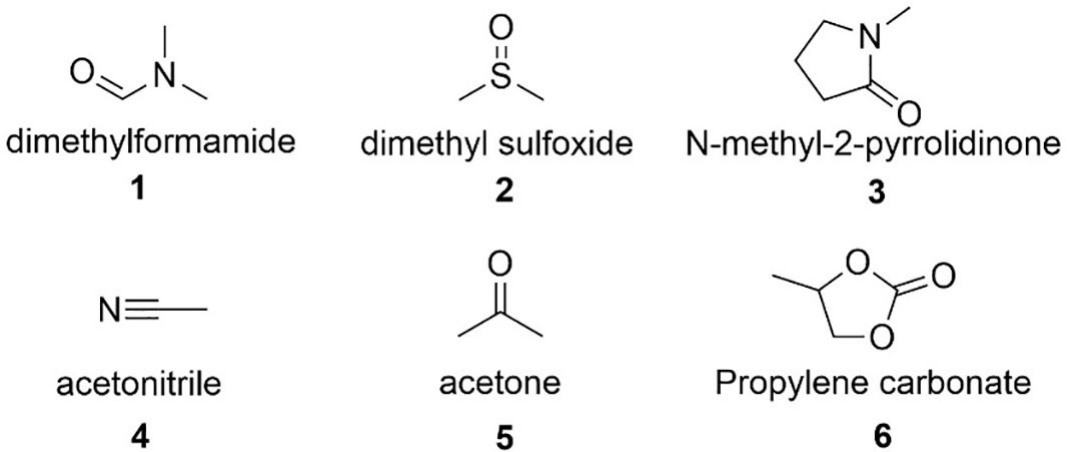
Structure of common polar aprotic solvents.

Polar aprotic solvents are widely used in organic chemistry due to their excellent ability to dissolve a range of materials, including salts, making them especially versatile as reaction media. This solvation capacity arises from high dielectric constants, which enable the solvents to stabilise charged species and effectively solvate ions and polar molecules, even facilitating the dissolution of ionic compounds. Unlike protic solvents, dipolar aprotic species do not engage in hydrogen bond donation, which prevents strong hydrogen bonding interactions between solvent and nucleophiles, therefore allowing them to remain more reactive in solution, aiding solvation without interfering with the reactivity [10]. In addition to their physicochemical properties, these solvents are often low‐cost and readily available, making them an attractive reaction medium. Common dipolar aprotic solvents are dimethylformamide (DMF) **1**, dimethyl sulfoxide (DMSO) **2**, *N‐*methyl‐2‐pyrrolidinone (NMP) **3**, acetonitrile (MeCN) **4**, acetone **5** and propylene carbonate (PC) **6** (Figure 1).

According to a survey of publications reported in Organic Process Research and Development (OPR&D), the most popular dipolar aprotic solvents are DMF, acetonitrile, DMSO and NMP [11]. The 2020 analysis across three representative journals (OPR&D, the Journal of Medicinal Chemistry and Angewandte Chemie) showed that dipolar aprotic solvents comprised 17%–20% of all solvents employed, demonstrating their consistent importance across different areas of chemical research, from process to medicinal chemistry and fundamental organic synthesis [8].

It is well known that conventional organic solvents pose significant environmental, health and safety (EHS) concerns. Specifically, solvents such as DMF, DMSO and NMP (all mentioned above) are classified as hazardous due to their toxicity, carcinogenicity and environmental persistence [12, 13]. These solvents contribute to air pollution through VOC emissions, contaminate water sources and represent occupational health risks to workers via inhalation and dermal exposure [14]. Additionally, the energy‐intensive production and disposal of these solvents contribute to the carbon footprint of chemical processes [15].

Given these concerns and the large volumes of solvent used across the broad chemistry sector, efforts to transition away from traditional solvents have increased. Green solvents offer reduced toxicity, lower environmental impact and enhanced safety profiles whilst maintaining comparable or superior performance in chemical reactions [16].

### 1.2 Properties of Propylene Carbonate

Propylene carbonate is a colourless, biodegradable liquid. It is also non‐corrosive and economically viable, which makes it suitable for large‐scale use [17, 18]. Its cyclic and strongly polar structure makes it a sustainable medium for chemical transformations that require a stable polar environment. As shown in Table 1, PC exhibits a dielectric constant of *ε* = 64 at 20 °C, providing an excellent capacity for dissolving ions and supporting polar reactions. In comparison, other common polar aprotic solvents, such as acetonitrile (MeCN) and acetone, possess significantly lower dielectric constants (*ε* = 37 and *ε* = 21, respectively). Although dimethyl sulfoxide (DMSO) also exhibits a high dielectric constant (*ε* = 46), its toxicity profile and relatively low boiling point limit its wide and safe application.

**TABLE 1:** Propylene carbonate and common organic solvents properties (table adapted from H. Sneddon and co‐workers) [8].

	GSK^a^	B.p., °C^b^	M.p., °C^b^	Vapour pressure (mmHg, 20 °C)^b^	$E_{T}^{N}$ c	Dielectric constant^c^	Hazard class^b^	Biodegradability^b^
Propylene carbonate (PC)		243	−55	0.04	0.472	64	Health hazard	Readily biodegradable
Acetonitrile (MeCN)		82	−44	73	0.460	37	Health hazard/flammable	Moderately
Dimethylsulphoxide (DMSO)		189	18	0.42	0.444	46	Health hazard	Moderately
Acetone		56	−95	188	0.355	21	Health hazard/flammable	Readily biodegradable
Cyrene		227	−20	0.21	0.333	37 [19]^d^	Health hazard	Biodegradable [20]
*N‐*Butyl‐2‐pyrrolidinone (NBP)		241	−75	0.1	0.323	32 [8]^d^	Health hazard	Biodegradable [21]
Dimethyl carbonate (DMC)		90	4	55.33	0.232	3 [22]	Flammable	Biodegradable [23]
Dichloromethane (DCM)		40	−95	435	0.309	9	Health hazard/irritant	Poorly biodegradable
Sulfolane		285	26	0.006	0.410	43 [24]	Health hazard	Moderately [25]
*N*,*N‐*Dimethylformamide (DMF)		163	−60	2.7	0.386	37	Health hazard/serious health hazard	Readily biodegradable

a GSK: GlaxoSmithKline.

b B.p, m.p., vap. pressure, hazard class and biodegradability obtained from data sheets and safety datasheets (National Centre for Biotechnology Information).

c Dielectric constants (@20 °C) and relative polarity from Organic Chemistry Data (Properties of Solvents Used in Organic Chemistry).

d Dielectric constants (@25 °C).

Another remarkable property of PC is its high boiling point (243 °C), which, combined with its low vapour pressure, prevents the generation of toxic vapours, making it suitable for transformations that require high temperatures or extended reaction times. These characteristics not only reduce solvent loss but also enhance safety by reducing the flammability risk. By contrast, halogenated solvents with low boiling points, such as DCM (40 °C), and high volatility present increased risks of emission and inhalation. Although DMSO presents a moderate boiling point (189 °C), it remains inferior to PC across multiple parameters, as illustrated in Table 1.

PC offers good miscibility with a broad range of organic solvents and moderate solubility in water, facilitating both reaction work‐up and product isolation. These properties align with the principles of Green Chemistry, particularly those related to safer solvents, energy efficiency, reduced hazard and potential CO_2_ valorisation. PC shows low aquatic toxicity (LD_50_ > 29 g/kg in rats), underscoring its favourable environmental profile. Nevertheless, some practical limitations must be acknowledged: the high boiling point can hinder the removal of organic products during work‐up, and PC is partially miscible with water, which may complicate aqueous extractions. Furthermore, its relatively high viscosity can slow mass transfer in some transformations, and it is susceptible to hydrolysis under stronger basic conditions, which requires consideration in the reaction design. These challenges will also be discussed in greater detail throughout this review.

Overall, the advantageous physical and chemical properties, combined with sustainable synthesis (see the next section), establish propylene carbonate as an excellent green solvent for modern organic synthesis.

### 1.3 Propylene Carbonate Synthesis

**SCHEME 1:**
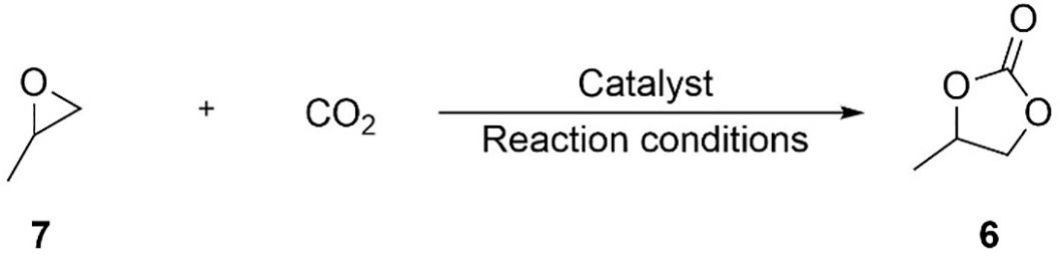
General scheme of propylene carbonate synthesis.

Commercial propylene carbonate production focuses on CO_2_ valorisation via a 100% atom‐economical cycloaddition with propylene oxide (Scheme 1). Recent developments in PC synthesis still prioritise CO_2_ utilisation, with advances made using milder reaction conditions and improvements in catalytic efficiency, leading to the development of more sustainable and efficient methods.

**SCHEME 2:**
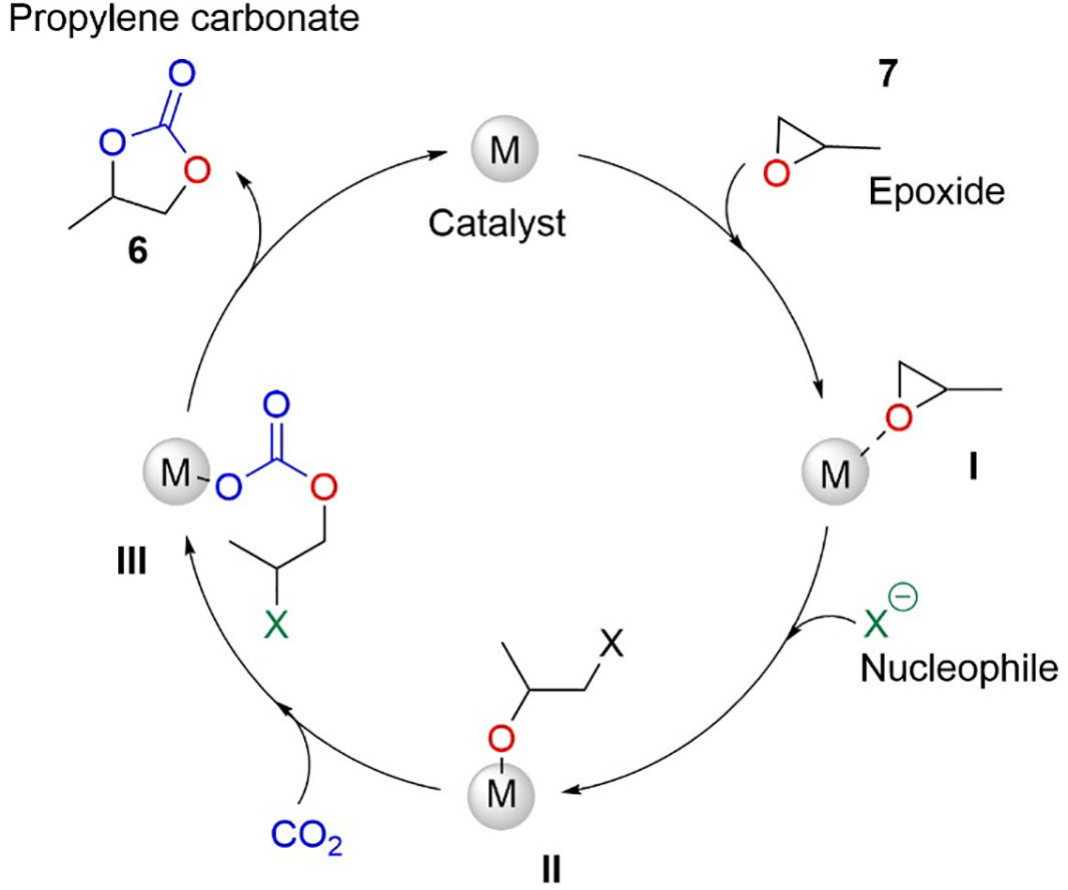
Reaction mechanism of cyclic carbonate synthesis.

The process of propylene oxide and CO_2_ cycloaddition is generally based on a catalytic system that combines a Lewis acidic metal (**M**, e.g. Zn^2+^, Al^3+^, and Cr^3+^) with a nucleophilic species (**X** ^−^, e.g. Cl^−^, Br^−^, and I^−^). As shown in Scheme 2, the metal binds to epoxide **I**, therein activating it towards nucleophilic attack and subsequent ring opening of epoxide **II**. The next step involves the insertion of CO_2_ into the metal–alkoxide bond, leading to the formation of a hemicarbonate intermediate **III**. Finally, it undergoes ring‐closing to afford the cyclic carbonate **6** (Scheme 2).

Although the cycloaddition of CO_2_ with epoxides proceeds with a 100% atom economy, the catalytic process has some drawbacks that detract from the green focus. Conventional catalytic routes typically rely on metal‐based homogeneous catalysts, which, whilst effective, present several disadvantages. These include the use of toxic metals, which often contaminate the product, whilst the recovery and recyclability of the metals are difficult. Additionally, the process often requires harsh conditions, resulting in higher energy consumption and costs [26]. To address these limitations, special attention has been given to developing new and more sustainable catalytic processes.

Given that the transformation is highly dependent on the synergy between the Lewis acid and the nucleophile and, in some cases, on the presence of a co‐catalyst for improved selectivity, this has been the subject of extensive research that has led to the development of several highly efficient catalytic systems. Among these systems, both homogeneous and heterogeneous catalysts have been employed. Homogeneous catalysts provide greater contact between the active species and reactants, resulting in enhanced reaction rates, whereas heterogeneous systems facilitate product isolation and catalyst recovery [27]. Following the sustainable approach emphasised throughout this review, here, we provide an overview of recent homogeneous and heterogeneous catalysts that have demonstrated effectiveness in CO_2_ cycloaddition with epoxides whilst maintaining environmentally sustainable profiles. As such, Table 2 illustrates selected recent examples for the synthesis of PC using a range of different catalytic systems, both homogeneous and heterogeneous, separated by the catalyst type.

**TABLE 2:** Reported homogeneous and heterogeneous catalysts for PC synthesis from CO_2_ and PO with homogeneous and heterogeneous catalyst systems.

Type of catalysts	References	Catalyst	Co‐catalyst	Reaction conditions				Yield, %	TON^a^	TOF, h^−1^ a	Reactor
				Reaction parameters	Temperature, °C	Time, h	CO_2_ pres., MPa				
*Organocat.*	[28]	Cholic acid‐based bis‐primary amine	TBAI	PO 0.2 mL, catalyst 1 mol%	80	24	0.1	64	64	2.7	Batch
*Ionic liquids* (*ILs*)	[29]	[Hmim]_2_[ZnBr_4_]	Free	PO 1.5 mL, molar ratio of zinc to epoxide 0.2	25	5	0.1	99	5	1	Batch
*Deep eutectic solvents* (*DES*)	[30]	2:1 molar ratio ChI/CA	Free	PO 34.5 mmol, catalyst 3 mol%	70	3	0.5	98	32.5	10.8	Batch
*Supported ionic liquids* (*SILCs*)	[31]	MCM‐22‐CPTES‐[AeMIM] [Zn_2_Br_5_]	Free	Molar ratio of CO_2_/PO of 3:1	130	50	2.0	62	N/A^b^	N/A^b^	Fixed bed
*Supported polymers*	[32]	Quaternary ammonium salt IL‐based ionic porous polymer (DVB‐HTA)	Free	PO (16 mmol), DVB‐HTA (0.035 mmol)	120	6	1.2	93	425	70.9	Batch
*M* *etal* * organic frameworks* (*MOFs*)	[33]	MIL‐100(Sc)	TBAB	PO (165 mM), catalyst (42 mg), co‐catalyst (8.4 mM)	100	5	0.5	~58%	28^c^	N/A^b^	Flow
*Zeolites*	[34]	Ti‐Beta‐3%	KI	PO (20 mmol), catalyst (50 mg), additive (40 mg)	80	3	1.5	55%	119^c^	~357	Batch

Abbreviations: TBAB, tetrabutylammonium bromide; TBAI, tetrabutylammonium iodide.

a Calculated from data reported in the paper.

b Cannot be calculated due to reactor type or insufficient data.

c Value given by the authors.

#### 1.3.1 Organocatalysts (Metal‐free, H─Bond Donor, Organic Bases)

These catalysts are organic compounds (e.g. nitrogenous bases and hydrogen bond donors), thus replacing the role of metals in the conventional mechanism as they promote epoxide activation [35]. This eliminates metal contamination and reduces the costs associated with traditional metallic catalysts. The mechanism by which they operate includes hydrogen bond activation [36, 37], with catalysts such as thioureas and squaramides [38, 39]; organic bases, including DBU and TBD, which operate through Brønsted acid–base catalysis [40, 41]; or the formation of covalent intermediates [42, 43]. Recent studies have shown good activity and, in some cases, recyclability [28, 44, 45].

#### 1.3.2 Ionic Liquids (ILs) and Deep Eutectic Solvents (DES)

Another option for homogeneous catalysts is ionic liquids, which are salts in a liquid state near room temperature, with low melting points and entirely composed of organic cations and anions. These provide high thermal and chemical stability, low vapour pressures, and an easily adjustable structure, allowing for optimisation of catalyst activity and selectivity by adjusting the ions [27]. Furthermore, like an organocatalyst, they offer the advantage of being metal‐free, avoiding potential contamination. Another benefit of these catalysts is that they can be recycled multiple times without a significant loss of activity, though their cost is higher than that of simple salts like TBAB. Some commonly used ionic liquids include imidazolium ([BMIM]Br, [EMIM]I), triazolium ([BnTriMe]Br), pyridine, or bifunctional compounds with ─OH, ─NH_2_ or ─COOH groups, which incorporate hydrogen‐bonding groups that enhance dual activation. ILs have demonstrated high yields (>90%), especially in optimised systems and in flow reactors [46, 47, 48].

On the other hand, DES are eutectic mixtures composed of a hydrogen‐bond acceptor (generally ammonium or phosphonium salts) and a hydrogen‐bond donor (such as urea or organic acids). As ILs, deep eutectic solvents are thermally stable, have low vapour pressure, and are non‐flammable; however, they offer additional advantages: they are more economical, biodegradable, and less toxic [49]. Some of the most commonly used DES combinations are bifunctional, which combine a halide (nucleophile) with a hydrogen bond donor (such as an acid or alcohol). This includes biomass‐derived DES such as choline chloride (ChCl)/lactic acid or betaine/urea or DES + co‐catalyst (e.g. ZnBr_2_ or TBAB), which allows higher yields under mild conditions (<80 °C, <5 bar) [50, 51].

Whilst homogeneous catalysts provide excellent activity, heterogeneous catalysts offer additional advantages, particularly in terms of catalyst recovery and process efficiency, making them particularly attractive from both an industrial and sustainability perspective. Among the numerous systems available, certain heterogeneous catalysts have attracted considerable attention recently.

#### 1.3.3 Supported Ionic Liquids (SILCs) and Supported Polymers

These types of catalysts consist of a thin layer of ionic liquids immobilised on a porous support (such as silica or alumina). In this case, the ionic liquid provides the nucleophilic anion (Cl^−^, Br^−^, and I^−^), whilst the support offers stability and dispersion of active sites [27, 52]. By immobilising ionic liquids on solid supports, SILCs can operate as heterogeneous catalysts, overcoming the viscosity limitation of conventional ionic liquids. They therefore exhibit the high activity and selectivity of homogeneous catalysts combined with the ease of separation and recyclability of heterogeneous catalysts [53]. Supported polymers, on the other hand, involve covalent bonding; these are materials where a catalytically active species (metal centre, organometallic complex, or organocatalyst) is immobilised or covalently bonded to an insoluble polymer matrix (typically polystyrene, polyacrylate or polyethylene glycol) [54]. Similar to supported ILs, supported polymers maintain the activity and selectivity of a homogeneous catalyst whilst facilitating its recovery and reuse [55]. Some examples of the materials used for the cycloaddition of CO_2_ with epoxides are polymers with quaternary ammonium salts immobilised in polystyrene resins or porous polymers with bifunctional sites containing amino groups and Lewis acid sites [32, 56, 57].

#### 1.3.4 MOFs and Functionalised Zeolites

MOFs are porous crystalline materials with active sites (metallic or organic) that can activate both epoxides and CO_2_. Composed of metal ions coordinated with organic ligands, they present 3D structures with large surface areas, allowing them to support bifunctional active sites [27]. In the cycloaddition with epoxides, MOFs act in two main ways: epoxide activation occurs at the Lewis acidic metal nodes, and CO_2_ activation occurs at ligands functionalised with amino groups or halides that can activate CO_2_ and facilitate its insertion [58]. This cooperative mechanism, through the metal node and the organic ligand, allows for good conversions at moderate temperatures and pressures [59, 60].

Similarly, zeolites are microporous aluminosilicates with very well‐defined crystalline structures. They also consist of large pores that can be easily adjusted as zeolites can have many functional sites, which can therefore act as Brønsted acid or Lewis acid/base sites, providing great capacity for CO_2_ absorption, both inside the pores and on the surface of the material [61]. Zeolites are also chemically and thermally stable, allowing them to be used in high‐temperature processes and to be easily reactivated by combustion, pyrolysis, or thermal treatments [62]. Studies indicate that they exhibit high yields and selectivity, even under mild conditions and solvent‐free conditions [63, 64].

Organocatalysts, ILs and DES are gaining strong attention for their sustainability profile and metal‐free character [65, 66, 67]. However, heterogeneous systems, particularly metal oxides and supported catalysts, continue to lead in industry and will likely continue to consolidate as the main method of PC production, due to their technological maturity and industrial viability [68, 69].

To provide a more quantitative perspective on the performance of these systems, two key metrics warrant consideration: turnover number (TON) and turnover frequency (TOF). TON represents the total number of substrate molecules converted per active catalytic site over the course of the reaction, serving as a measure of overall catalyst productivity, whilst TOF expresses this as a rate, the number of turnovers per unit time (h^−1^), thus reflecting intrinsic catalytic activity. Together, these parameters allow for a meaningful comparison of catalytic systems independent of catalyst loading and are particularly valuable when assessing the efficiency and potential scalability.

As illustrated in Table 2, the performance of representative systems varies considerably across catalyst types. Organocatalysts, such as cholic acid‐based bis‐primary amines with TBAI as co‐catalyst [28], achieve yields of 64% under mild conditions (80 °C, 0.1 MPa), with TON and TOF values of 64 and 2.7 h^−1^, respectively. Ionic liquids, exemplified by [Hmim]_2_[ZnBr_4_] [29], demonstrate notable performance under near‐ambient conditions (25 °C, 0.1 MPa), reaching 99% yield, although with a modest TOF of 1 h^−1^, suggesting slower intrinsic kinetics despite the high conversion. DES systems, such as ChCl/CA (2:1 molar ratio) [30], deliver a 98% yield at 70 °C and 0.5 MPa, with a TON of 32.5 and TOF of 10.8 h^−1^, highlighting their competitiveness under mild and sustainable conditions. Among heterogeneous systems, supported polymers (e.g. DVB‐HTA) [32] stand out with the highest TON (425) and TOF (70.9 h^−1^) among the systems examined, operating at 120 °C and 1.2 MPa, which highlights the advantage of dispersing active sites on solid supports. MOFs (MIL‐100(Sc) with TBAB co‐catalyst) [33] and zeolites (Ti‐Beta‐3% with KI) [34] afford moderate yields (~58% and 55%, respectively), with the zeolite system achieving a TON of ~119 at 80 °C and 1.5 MPa, and both demonstrating compatibility with batch and flow configurations.

When comparing these systems, no single catalyst emerges as universally superior; rather, the optimal choice depends on the specific requirements of the reaction. For processes prioritising mild conditions and high yields, [Bmim][ZnBr_4_] and DES systems are highly effective, with the latter offering the additional benefits of biodegradability and low toxicity. However, in terms of catalytic efficiency, supported polymers provide the most significant results, combining high TON and TOF values with excellent recyclability, which are essential factors for industrial scalability. Meanwhile, zeolites and MOFs offer moderate yields but remain valuable due to their thermal stability and suitability for continuous flow processes. Ultimately, selecting the right system requires balancing factors such as reaction scale, sustainability goals, and ease of product isolation.

## 2 Propylene Carbonate as a Green Solvent in Organic Synthesis

### 2.1 Metal‐Catalysed Cross‐Coupling Reactions

**SCHEME 3:**
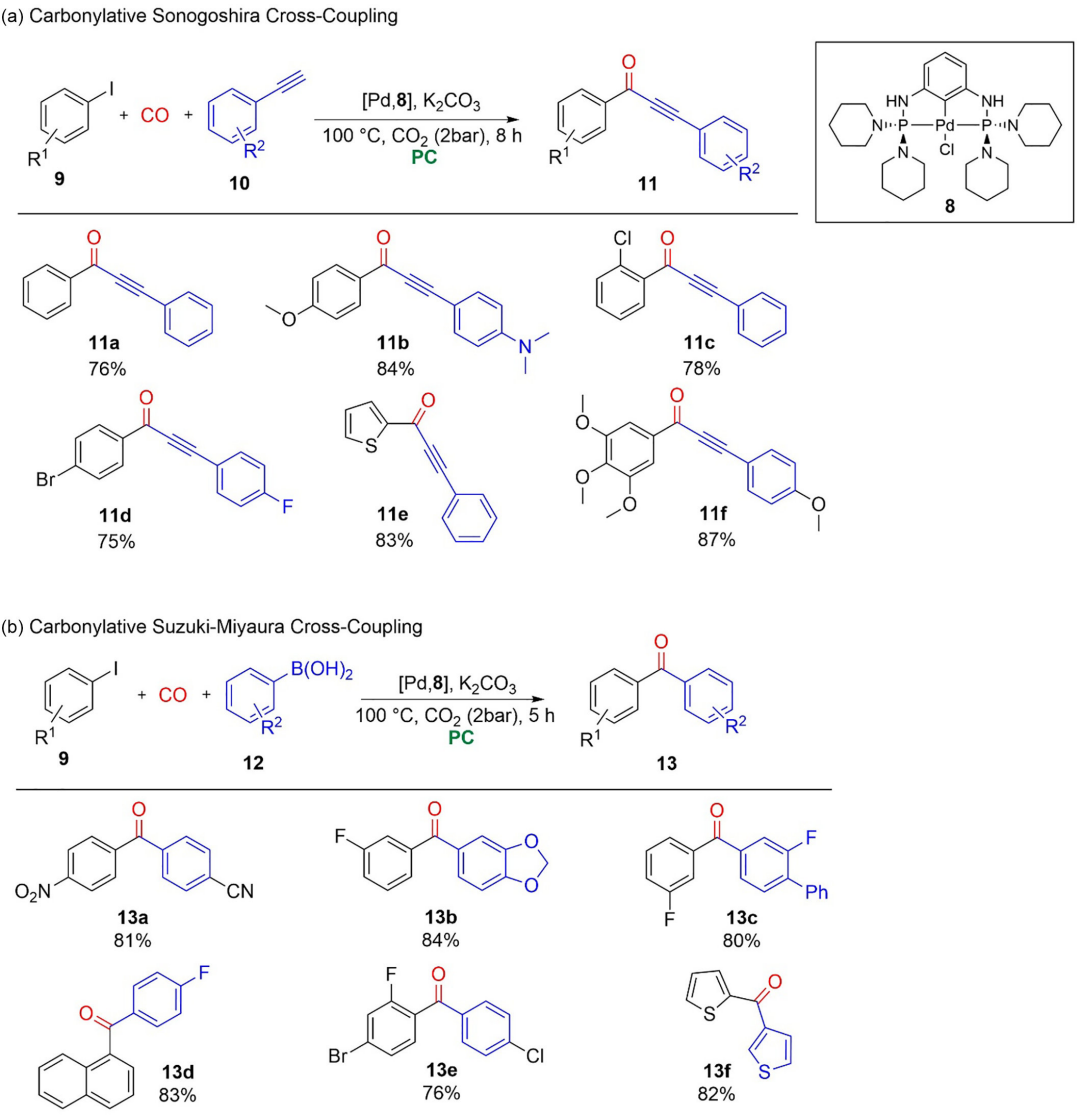
Palladium aminophosphine pincer complex‐catalysed carbonylative (a) Sonogashira and (b) Suzuki–Miyaura cross‐coupling reactions.

Bhanage and co‐workers [70] explored the efficacy of PC in Carbonylative Sonogashira (CS) and Suzuki–Miyaura (CSM) cross‐coupling reactions catalysed by palladium aminophosphine pincer complexes, traditionally dominated by hazardous solvents such as DMF, toluene or dioxane (Scheme 3). The optimised protocol uses an aminophosphine pincer complex {[C_6_H_3_‐2,6‐(NHP{piperidinyl}_2_)_2_]Pd(Cl)} (**8**) and benefits from being able to employ potassium carbonate as an inexpensive and moisture‐tolerant base.

Whilst PC achieved good yields, conversions and selectivity that were comparable to traditional media (toluene, DMF, dioxane, ethylene carbonate and anisole) across a broad substrate scope, marked superiority in catalytic efficiency was observed, as evidenced by significantly enhanced turnover numbers (TON) and turnover frequencies (TOF). Beyond the inherent favourable environmental profile and safety advantages—specifically a non‐carcinogenic nature—compared to restricted solvents like DMF and dioxane, the performance of PC in these cross‐coupling reactions is primarily driven by the high dielectric constant. This physical property facilitates CO solubility, which is particularly important in carbonylative pathways, where dissolved CO concentration is critical, as a higher molarity in the medium ensures the rapid formation of the acyl‐palladium species, effectively outcompeting the direct interception of the boronic acid (Scheme 3b), which would otherwise lead to the non‐carbonylative Suzuki by‐product. This mechanistic advantage is evident in the superior reaction selectivity of PC over DMF. Whilst in the CS reaction, both PC and DMF achieved similarly high product selectivity (96% in PC vs. 91% in DMF), in the CSM reaction, however, the difference was much larger; PC afforded 94% selectivity compared to just 19% in DMF. Whilst both solvents are polar aprotic, the amide carbonyl group in DMF can coordinate to palladium, which prevents effective CO insertion in the carbonylative reactions. In contrast, PC doesn’t compete for Pd coordination, proving to be a robust medium in which carbonylation can occur. Elsewhere, the high dielectric constant of PC promotes effective solvation of the K_2_CO_3_ base and stabilises the ionic intermediates of the catalytic cycle. This stabilisation prevents palladium deactivation, maintaining TON values between 10^5^ and 10^7^. This catalytic efficiency was demonstrated by using ultra‐low catalyst loading (10^−4^ mol% Pd), where PC maintained high performance in both transformations: 52% conversion and 94% selectivity for CS and 91% conversion and 95% selectivity for CSM. This is a level of efficiency that cannot be matched by traditional solvents such as toluene.

Finally, the high boiling point of PC offered operational safety and kinetic consistency. With reaction temperatures (100–120 °C) significantly below the boiling point (243 °C), PC exerts negligible vapour pressure (<1 mmHg), ensuring that the total system pressure in the autoclave corresponds almost exclusively to the CO partial pressure, allowing for precise control and reproducible reaction kinetics. In contrast, more volatile solvents such as toluene (b.p. 111 °C) and dioxane (b.p. 101 °C) contribute substantially to the vapour pressure in the headspace, which not only represents a safety risk but also dilutes the effective partial pressure of CO, potentially compromising the reaction rate and selectivity.

**SCHEME 4:**
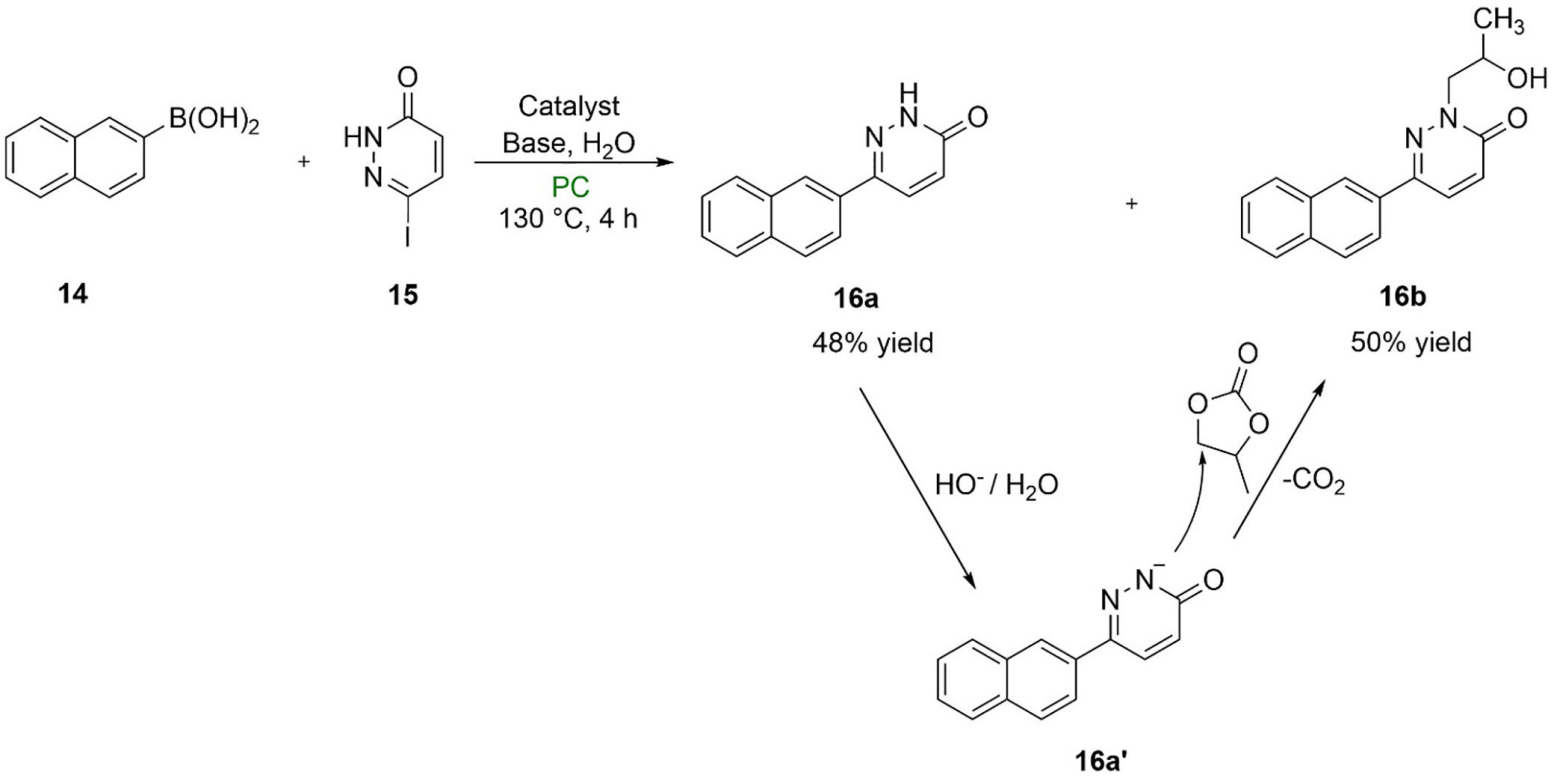
Suzuki–Miyaura reaction between 6‐iodopyridazin‐3(2H)‐one **15** and 2‐naphthaleneboronic acid **14**.

In 2019, Czompa and co‐workers [71] investigated the use of PC as a solvent for the Pd‐catalysed Suzuki–Miyaura reaction in the synthesis of biaryls (Scheme 4). Once again, PC proved to be an effective medium for this reaction, affording the product biaryls in typically good yields (>65%). Here, the high boiling point of PC facilitated the use of microwave irradiation (MW) as a heating source, which significantly reduced reaction times from 7 h (in an oil bath) to 1 h and without thermal decomposition.

**SCHEME 5:**
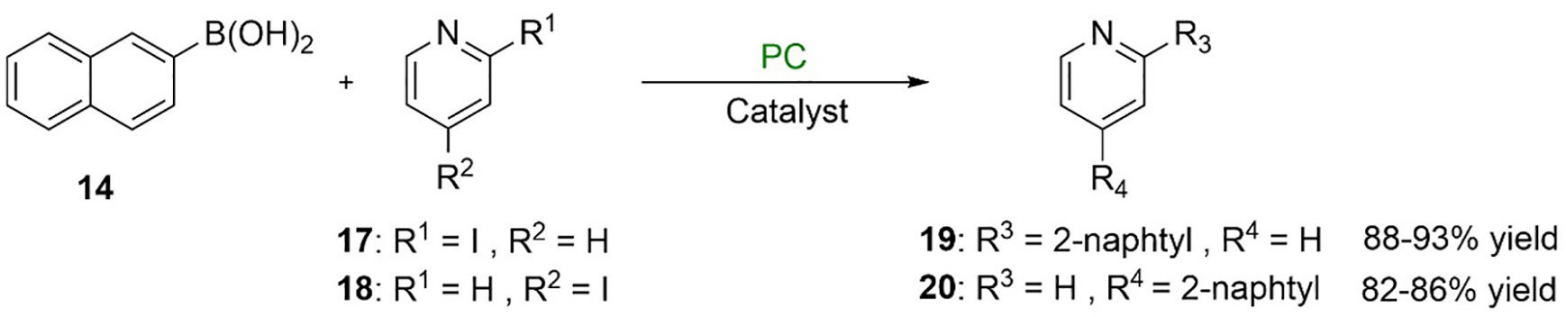
Synthesis of 2‐ and 4‐(naphthalen‐2‐yl)pyridine (**19,20**).

Interestingly, the study revealed that PC is not always inert under the basic conditions of the Suzuki reaction. Specifically, when a substrate (**15**) contains an acidic N─H group (p*K*_a_ ≤ 12), the base promotes deprotonation, and the resulting anion (**16a’**) attacks the electrophilic carbonyl group in PC, leading to ring opening and the formation of a 2‐hydroxypropyl adduct (**16b**). This side reaction represents a limitation for this reaction, where acidic N─H bonds are present, as the two alternative reaction pathways (carbonate ring opening before or after Suzuki coupling) mean that the side reaction cannot be avoided by a faster Suzuki reaction, since the coupled product itself is also susceptible. It was also demonstrated that iodopyridines **17** and **18** do not form side‐products (Scheme 5), as they lack acidic N─H protons. The clear structure‐reactivity correlation serves as a critical guide for synthetic chemists: PC is compatible with non‐acidic *N*‐heterocycles but incompatible with the lactam‐type N─H substrates used in this study under basic aqueous conditions.

**SCHEME 6:**
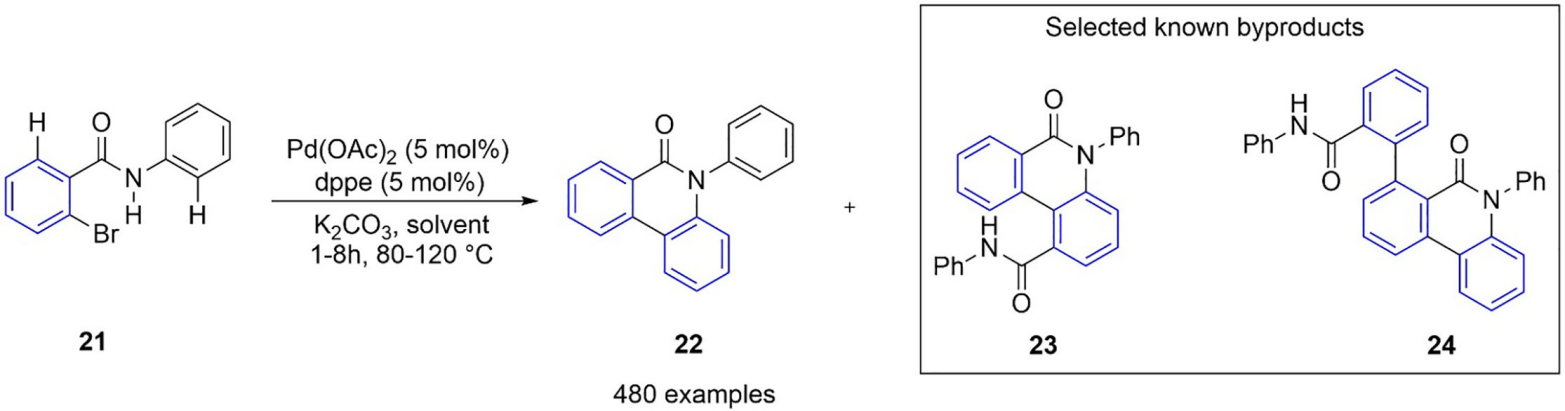
Synthesis of 5‐phenylphenanthridin‐6(5*H*)‐one **22** from 2‐bromo‐*N*‐phenylbenzamide **21**.

The role of polarity in influencing both conversion and by‐product formation in Pd‐catalysed reactions has been systematically explored by Clarke et al. [72], who conducted an extensive screening of 480 reaction profiles across 8 solvents, 4 temperatures, and at 4 time points for the intramolecular C─H arylation of 2‐bromo‐*N*‐phenylbenzamide **21** (Scheme 6). The primary finding was that PC and DMF consistently delivered high conversion to the target product **22**, with all other solvents underperforming for different reasons (Table 3). MeCN and butyronitrile (BuCN) showed reduced conversion and accumulation of specific by‐products, which the authors associate with competitive Pd coordination by the nitrile group, which diverts the catalytic cycle away from the productive pathway. On the other hand, toluene and Bu_2_O gave very low conversion to product **22** due to the low polarity and subsequent inability to stabilise the charge‐separated ionic intermediates of the catalytic cycle. Lastly, methyl ethyl ketone (MEK) acted as a reactive medium rather than an inert solvent, serving as a substrate for the Pd‐catalysed α‐arylation.

**TABLE 3:** Solvent‐dependent product distribution in the synthesis of **22**, indicating the frequency of high‐yield observations across 16 experiments per solvent.

Product	Note	Solvents							
		MeCN	Bu_2_O	Toluene	DMF	MEK	PC	BuCN	Bu_2_O:DMF (9:1)
**21**	Starting material (unreacted)	2	16	3	/	1	/	2	1
**22**	Target product	/	/	/	16	/	16	/	7
**23**	DMF‐specific by‐product	/	/	4	16	/	/	/	/
**24**	PC‐specific by‐product	/	/	/	9	/	14	/	/

*Note*: Colour scale: number of experiments (out of 16) in which a high quantity of the indicated product was observed.
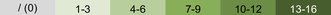

The physicochemical properties of PC enhance its performance for this type of reaction, as it is a good medium to stabilise charge‐separated transition states throughout the catalytic cycle. However, despite the comparable performance of PC and DMF, the two solvents produced markedly different by‐product profiles, as illustrated in Scheme 6 and Table 3. The analysis reported by Clarke et al. shows that PC selectively favours the formation of compound **24**, present in 14 out of 20 PC experiments, whereas DMF produces compound **23**, observed in 16 out of 20 DMF experiments. The authors proposed that the selectivity of PC towards by‐product **24** can be attributed to the high‐polarity environment, where a C─H activation of the already formed **22** occurs by a Pd(II) intermediate. This is important from a synthetic perspective, as, unlike by‐product **23**, which is formed by diverting a catalytic intermediate before **22** is ever produced, **24** is generated downstream, meaning that it directly consumes the target product, resulting in lower yield and a more challenging purification, since **22** and **24** are structurally similar, making their chromatographic separation challenging. However, the authors do not consider the accumulation of **24** in PC as a limitation but rather as a potential discovery opportunity, suggesting that if **24** were itself the synthetic target, PC could be the preferred solvent.

Regarding practical considerations, the high boiling point of PC is advantageous in terms of thermal stability and negligible vapour pressure under the reaction conditions, allowing operation across the full temperature range studied (80–120 °C) without solvent loss or safety concerns. However, this property also introduces a significant practical drawback, especially in the recovery of the product, as solvent removal is more demanding and typically requires high‐vacuum distillation or other laborious techniques. The work‐up described in this study is an aqueous acidic wash and brine extraction, taking advantage of the full miscibility of PC with water; however, the authors do not address whether PC remains in the organic phase or discuss the potential contamination of the product with residual solvent, which are aspects that must be carefully considered. From a waste management perspective, it is worth noting that, unlike DMF, which requires special disposal protocols, PC waste streams are classified as non‐hazardous, representing a considerable advantage in industrial settings where disposal costs and regulatory compliance are very important.

**SCHEME 7:**
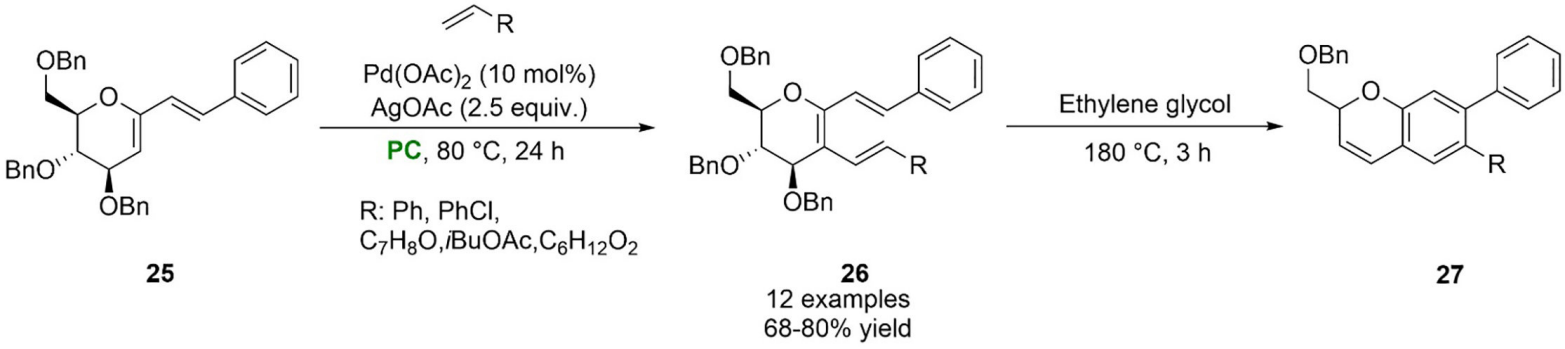
Pd‐catalysed cross‐dehydrogenative coupling for the synthesis of 1,2‐disubstituted glucals in PC.

A separate study by Maikuri et al. [73] investigated the synthesis of 1,2‐disubstituted glucals **26**, which could then be converted into 2*H*‐chromenes **27**, versatile heterocyclic building blocks for the synthesis of a large number of bioactive molecules (Scheme 7) [74]. The authors highlighted that the superior performance of PC in this and other similar Pd‐catalysed transformations is mainly driven by the high dielectric constant, which distinguishes it from the other dialkyl carbonate solvents investigated. Under identical conditions, PC achieved yields up to 80% across various substrates, significantly outperforming dimethylcarbonate (DMC, 35%) and diethyl carbonate (DEC, 0%), which have significantly lower polarities than PC (DMC = 3.1, DEC = 2.8, PC = 65). The high polarity of PC allows it to accelerate the catalytic process by stabilising transition states with high charge separation.

Beyond solvent polarity, the high boiling point of PC provides thermal headroom in the reaction vessel at 80 °C, unlike DMC, which operates near its boiling point under the reported conditions. Furthermore, as PC is a weak electron donor, it avoids competitive coordination to the Pd centre that would divert the catalytic cycle, a problem presented with nitrile‐based solvents in the study by Clarke et al.

From a sustainability perspective, in general, the substitution of a traditional DMF/DMSO solvent mixture with PC represents a meaningful step forward in terms of toxicity and waste classification. As discussed previously, PC is difficult to remove by standard evaporation apparatus; however, in this study, isolation of the product is reported via a simple aqueous extraction with ethyl acetate, followed by chromatographic purification. This implies that the PC is neither recovered nor reused, and it remains in the aqueous phase after extraction, rendering it a single‐use solvent in this example. Note that no comment is made as to whether any PC persists in the crude material prior to chromatography. Similarly, the study does not report specific data on PC recovery or recycling, which are important factors to consider with regard to sustainability.

**SCHEME 8:**
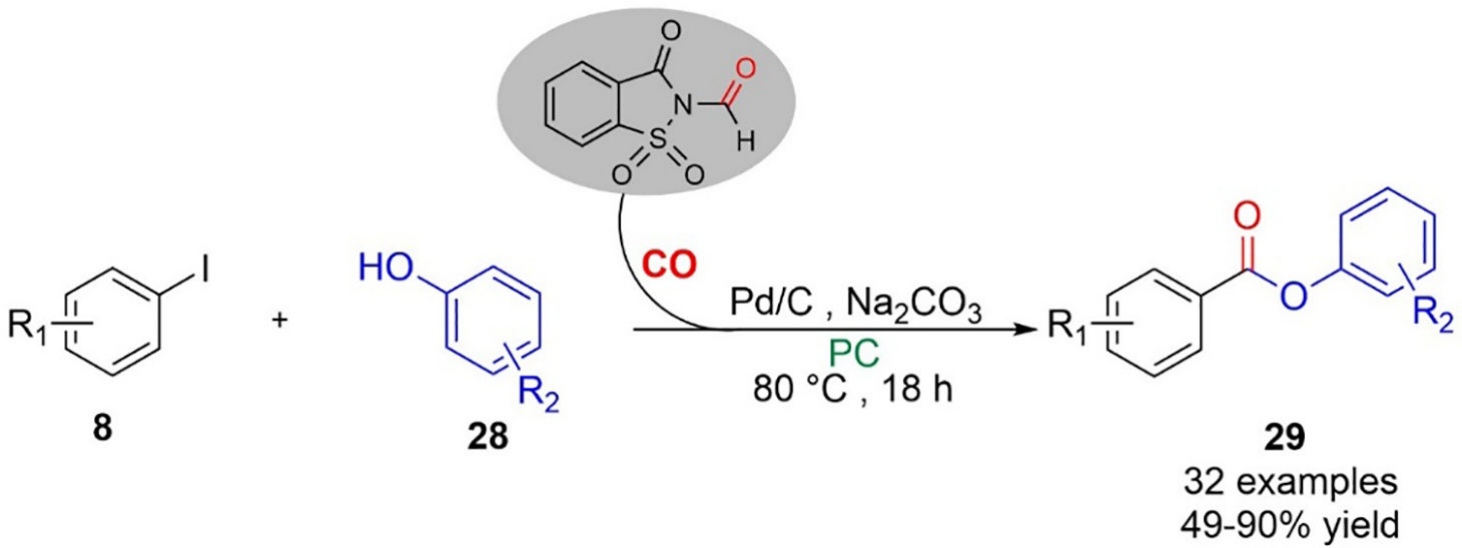
Synthesis of phenyl esters via Pd‐mediated carbonylation with *N*‐formylsaccharin as CO source.

Building upon their previous findings, in 2017, Bhanage et al. [75] extended the application of PC to the Pd/C‐catalysed phenoxycarbonylation of aryl iodides **8**, using *N*‐formylsaccharin as a safe CO source (Scheme 8). In this study, PC was selected as a sustainable alternative to DMF and DCM, the solvents conventionally employed in carbonylation reactions that employ *N*‐formylsaccharin. Furthermore, a solvent screening study confirmed that non‐polar solvents such as toluene and dioxane delivered low conversions (6% and 10%, respectively), consistent with their inability to stabilise the ionic intermediates within the catalytic cycle, a trend already discussed in the context of the preceding report. On the other hand, among the polar alternatives, PC outperformed ethylene carbonate (76% vs. 67% conversion), providing phenyl esters **29** in yields up to 90% when a ligand was also used. Importantly, PC maintained a high reaction efficiency (78% conversion) even under ligand‐free conditions, whereas a phosphine ligand (PPh_3_, 10 mol%) was required to achieve high conversion in DMF. The capacity of PC to facilitate the reaction under ligand‐free conditions is a significant finding, as it suggests that the more polar environment in PC not only stabilises charged intermediates in the catalytic cycle but may also facilitate the necessary molecular interactions at the Pd/C surface to sufficiently compensate for the absence of a stabilising ligand, reducing both cost and complexity of the reaction procedure.

In this report, the authors address key sustainability gaps. Notably, PC is compatible with Pd/C as a heterogeneous catalyst, enabling catalyst recovery through simple centrifugation. Pd/C was successfully recycled up to four times with only a marginal loss in conversion (from 77% to 73%), whilst ICP‐AES analysis confirmed palladium leaching below 0.1 ppm after the fourth cycle. The work‐up described is straightforward, including centrifugation to remove Pd/C, followed by an ethyl acetate wash and column chromatography, although, similar to the previous reports, it does not discuss the recovery or recycling of PC itself, which implies a single‐use disposal of the solvent.

A limitation across the literature employing PC is its separation from the final product; the high boiling point makes removal energy‐intensive and difficult to achieve via evaporation using standard equipment. Nevertheless, the waste profile of this carbonylation protocol is significantly more favourable, as the principal by‐product is saccharin, a non‐hazardous compound, and PC waste is classified as non‐hazardous, which represents a practical advantage in industrial settings.

**SCHEME 9:**
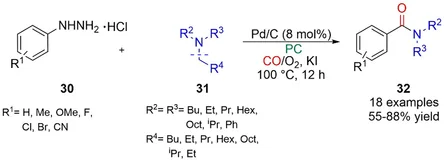
Pd‐catalysed aminocarbonylation reaction to produce aromatic amides.

The utility of PC as a reaction medium in Pd/C catalysed carbonylation transformations is further demonstrated by Kolekar and Bhanage [76] in a report on the oxidative aminocarbonylation of aryl hydrazines **30** via C─N bond activation (Scheme 9). The use of PC follows the same rationale as in the phenoxycarbonylation study discussed above, replacing the toxic and unsustainable conventional solvents for the oxidative carbonylation reactions, such as toluene, anisole and DMF. An informative solvent screen is presented in this study, covering a broad range of both non‐polar and polar aprotic solvents. Non‐polar solvents such as toluene and anisole gave moderate yields (72% and 68%, respectively), whilst conventional polar aprotic solvents including DMF, THF, dioxane, DMSO, MeCN and some binary mixtures all delivered inferior results, with DMF performing significantly lower at only 22% yield. This is particularly noteworthy given that DMF is one of the most widely used solvents for Pd‐catalysed carbonylations. In contrast, PC surpassed all other alternatives, giving an 87% yield under optimised conditions, which is again consistent with the ability to stabilise the charged intermediates generated within the catalytic cycle. The protocol proved amenable to a broad substrate scope, where aryl hydrazines (**30**) bearing both electron‐donating and electron‐withdrawing substituents reacted smoothly to afford tertiary amides **32** in up to 89% yield.

As established in the previous Bhanage group report using *N*‐formylsaccharin (see Scheme 8), the Pd/C catalyst could be recycled with only a marginal loss in activity; the five consecutive runs in this example represent an improvement on the four‐cycle recyclability reported in the phenoxycarbonylation study. However, consistent with the previous report, no data on PC recovery or recycling were detailed.

### 2.2 Oxidation Reactions

**FIGURE 2:**
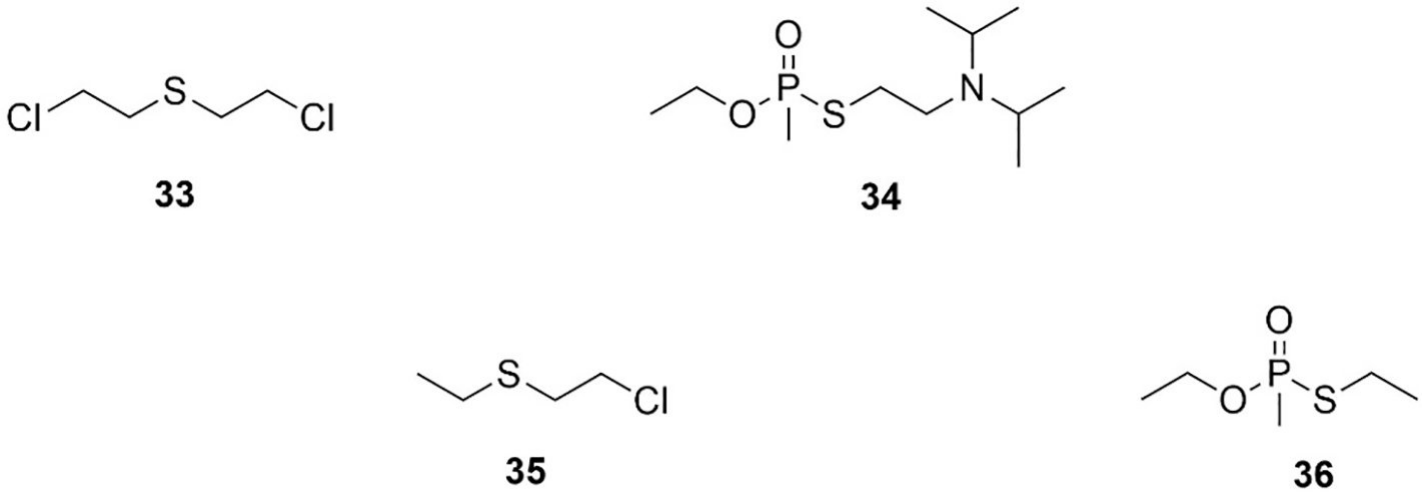
Sulphur‐containing CWAs and their simulants.

Work by Smolkin et al. [77] extends the application of PC to a different domain, the oxidative detoxification of sulphur‐containing chemical warfare agents (CWAs). Focussing on sulphur mustard (bis(2‐chloroethyl)sulphide, HD, **33**) and the toxic nerve agent, *O*‐ethyl‐*S*‐2‐(*N*,*N*‐diisopropylaminoethyl)methylphosphono thioate (**34**), the report uses electrophilic iodine reagents, I_2_ and *N*‐iodosuccinimide (NIS), in organic solvent/water mixtures (10:1 v/v) at room temperature (Figure 2). However, a considerable part of this study was conducted on CWA simulants, such as CEES **35** and DEMPT **36**. This is a particularly demanding application for any solvent, as it must simultaneously dissolve hydrophobic CWA substrates, support the electrophilic iodination mechanism, and remain chemically inert under mildly oxidative conditions. The key kinetic results for PC relative to the other solvents screened are summarised in Table 4.

**TABLE 4:** Reaction rates for the oxidation of CWAs (**33**,**34**) and their simulants (**35**,**36**) by I_2_ and NIS in different solvents.

Solvent	DN, Kcal/mol	CWAS ‐ *t*_1/2_, min				Simulants ‐ *t*_1/2_, min			
		HD, 33		34		**35**, CEES (**33** simulant)		**36**, DEMPT (**34** simulant)	
		I_2_	NIS	I_2_	NIS	I_2_	NIS	I_2_	NIS
MeCN	14.1	~92	<3	93	69	<3	<3	<3	<3
PC	15.1	~88	<3	27	30	<3	<3	<3	<3
Acetone	17	—	<3	320	44	<3	<3	<6	<3
THF	20	1080	4	43	60	7	<3	<4	<3
DMF	26.6	—	53	N/A	45	N/A	<3	<10	<3
DMSO	29.8	960	62	16	6	30	<3	<3	<3

*Note*: DN = donor number (lower DN correlates with faster HD **33** oxidation rates); — = incomplete conversion. Conditions: solvent/H_2_O 10:1 v/v, 11 equiv. oxidant, rt.

As shown in Table 4, PC gave consistently fast half‐life times (*t*_1/2_) across both oxidants and all CWA substrates (**33, 34**) and simulants (**35, 36**). For the oxidation of CEES and HD with I_2_, PC matched the performance of MeCN, which was the only other solvent to achieve full conversion and complete selectivity toward the non‐vesicant sulphoxide, with *t*_1/2_ <3 min for CEES **35** and *t*_1/2_ ~ 88 min for HD **33**, whilst higher donor number (DN) solvents such as THF (1080 min) and DMSO (960 min) performed poorly. With NIS, PC again achieved *t*_1/2_ < 3 min for both CEES **35** and HD **33**, with full conversion within 10–15 min, confirming NIS as the superior oxidant regardless of the solvent. In the oxidation of **34** NIS, PC delivered a *t*_1/2_ = 30 min, outperforming MeCN (69 min) and THF (60 min), with only DMSO showing a faster rate (6 min) due to a mechanistic pathway specific to thiophosphonate degradation that is unrelated to DN.

The most important parameter for the oxidation of mustard HD **33** is the DN of the solvent, rather than the dielectric constant, a key distinction from all the Pd‐catalysed reactions discussed in Section 2.1. The low DN of PC gives a decisive advantage by leaving the sulfonium intermediate accessible for attack by water. The authors recommend the PC/NIS combination as a powerful and more sustainable system for CWA decontamination, both in terms of its efficient performance and sustainability, with PC replacing the toxic solvents traditionally used.

A study by M. dos Santos et al. [78] extends the application of PC to Pd‐catalysed allylic oxidation of a range of alkenes: *cis*‐Jasmone **37**, cyclohexene **38**, limonene **39** and β‐citronellone **40** (Table 5). Using Pd(OAc)_2_/*p*‐BQ (10/50 mM) under 10 atm O_2_ at 60–80 °C in solvent mixtures containing 20–50 vol% acetic acid, the performance of PC was compared against dimethylcarbonate (DMC), methyl isobutyl ketone (MIBK) and pure acetic acid. PC proved to be faster than all the other green solvents tested, across virtually all substrates and even faster than pure HOAc, the conventional solvent for this reaction class.

**TABLE 5:** Oxidation of alkenes with molecular oxygen catalysed by Pd/BQ in different solvent systems.^a^

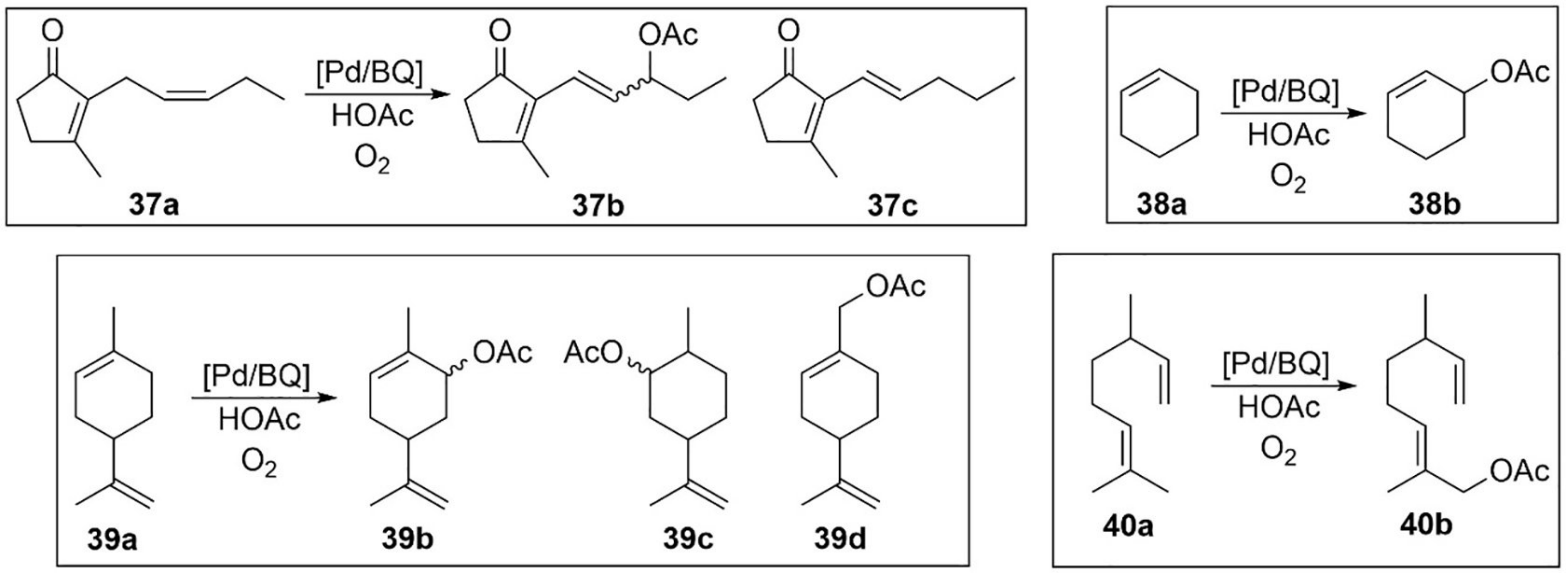				
Substrate	PC/HOAc	DMC/AcO	MIBK/AcOH	Pure Ac
	Conv. (%) | Sel. (%)| rate, mM/h	Conv. (%) | Sel. (%)| rate, mM/h	Conv. (%) | Sel. (%)| rate, mM/h	Conv. (%) | Sel. (%)| rate, mM/h
*cis*‐Jasmone, **37**	82 | **37b** (28), **37c** (70)| 164^b^^,^ c ^,^ d	95 | **37b** (90) | 36^e^^,^ f ^,^ g	90 | **37b** (87) | 72^e^^,^ c ^,^ g	97 | **37b** (82), **37c** (6) | 250^c^^,^ h
Cyclohexene, **38**	96 | **38b** (98)| 126^e^^,^ f ^,^ h	100 | **38b** (98) | 52^i^^,^ f ^,^ g	80 | **38b** (100) | 60^e^^,^ c ^,^ j	100 |**38b** (91) | 104^f^^,^ h
Limonene, **39**	96 | **39b** (12), **39c** (54), **39d** (28) |120^e^^,^ k ^,^ l	80 |**39b** (40),**39c** (47), **39d** (5) | 48^e^^,^ k ^,^ j	90| **39b** (38),**39c** (51),**39d** (4) | 70^m^^,^ k ^,^ g	96 | **39b** (33), **39c** (46),**39d** (10) | 80^k^^,^ h
β‐Citronellene, **40**	93 | **40b** (90) | 60^m^^,^ k ^,^ j	72 | **40b** (97)| 40^m^^,^ k ^,^ g	99 | **40b** (91) | 48^m^^,^ k ^,^ g	90 | **40b** (93) | 50^k^^,^ g

a Conditions: substrate = 0.20 M, Pd(OAc)_2_ = 10 mM, p‐BQ = 50 mM, gas phase – O_2_ = 10 atm.

b HOAc (vol%) = 50.

c *T* = 70 °C.

d Reaction time = 1 h.

e HOAc (vol%) = 20.

f *T* = 60 °C.

g Reaction time = 8 h.

h Reaction time = 4.

i HOAc (vol%) = 10.

j Reaction time = 6 h.

k *T* = 80 °C.

l Reaction time = 3 h.

m HOAc (vol%) = 30.

As mentioned elsewhere, the dielectric constant of PC plays a key role in the catalytic cycle by stabilising the ionic intermediates. It was observed that, for most substrates, the selectivity of the allylic oxidation in PC was higher or comparable to that observed in pure HOAc, representing a dual improvement in both the rate and selectivity. However, a substrate‐specific limitation was identified: for *cis*‐jasmone **37a**, the high polarity of PC promoted rapid substrate isomerisation to conjugated diene **37c** rather than the desired allylic acetate **37b**. These are important observations that highlight the typically advantageous nature of the enhanced dielectric properties of PC; however, they became detrimental in this case for substrates prone to isomerisation or with limited solubility in highly polar media. From a sustainability perspective, a minimum of 20–30 vol% AcOH was required to maintain substrate solubility in all PC formulations, which, whilst significantly reduced compared to a pure AcOH system, the generation of acetic acid waste remains a consideration.

**SCHEME 10:**
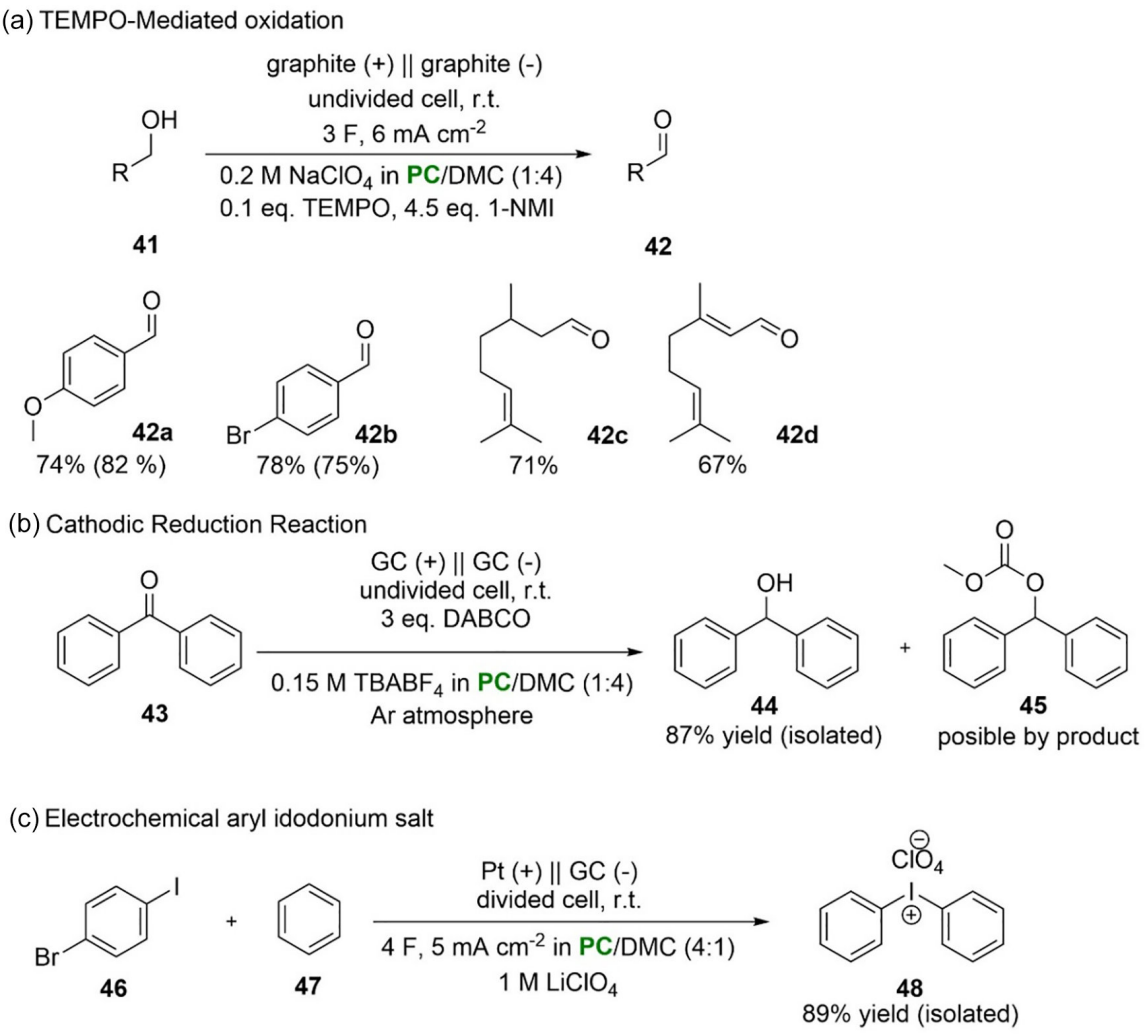
Anodic, cathodic and mediated electrochemical transformations in PC–DMC solvent mixtures.

In 2025, Prudlik et al. [79] extended the application of PC to organic electrosynthesis (Scheme 10). This is a particularly significant contribution as electrosynthesis requires a broader set of solvent properties than conventional reactions: the solvent must not only dissolve reactants and stabilise intermediates but also provide electrochemical stability, dissolve supporting electrolytes and maintain sufficient ionic conductivity. In this report, the authors propose a binary PC‐DMC system as a sustainable replacement for the conventional aprotic solvents most widely used in organic electrochemistry, MeCN, DMF and DCM.

The high dielectric constant of PC plays a different mechanistic role in the PC‐DMC system: rather than stabilising transition states, here, it governs ionic conductivity and electrolyte dissociation. Whilst pure DMC is unsuitable as a sole medium due to negligible conductivity, combining with PC at values above 20% w/w PC delivers acceptable conductivity. In addition, the electrochemical stability window (1:4) spans from −3.0 to +2.5 V versus Ag/AgNO_3_, which is comparable to that of MeCN and superior to both DMF and DCM, confirming its suitability for both oxidative and reductive transformations. A distinctive feature that is exploited across the model reactions is the tunability of different parameters, such as viscosity, polarity and conductivity, by varying the PC:DMC ratio.

The TEMPO‐mediated oxidation of primary alcohols **41** was more efficient when carried out in a green reaction medium than in traditional solvents (MeCN and DMF), highlighting its potential as a sustainable methodology (Scheme 10a). The results were quite similar when the oxidation was run in acetonitrile and neat PC (47% and 41% yields, respectively); a mixed solvent system of PC‐DMC afforded 74% yield under revised conditions, which improved to 82% with higher salt loading. This outperformed the corresponding reactions conducted in traditional solvents such as DMF, MeCN and DCM and the PC–DMC mixture delivered good yields among other primary alcohol substrates. This superior performance is attributed to a combination of the high polarity of PC—ensures adequate electrolyte dissociation and conductivity—and the high proportion of DMC (80% by volume), which reduces the viscosity of the system to maintain sufficient TEMPO diffusion rates.

For the cathodic reduction of benzophenone **43**, the PC‐DMC (1:4) solvent mixture delivered diphenylmethanol **44** in 87% isolated yield at a 5 mmol scale, surpassing the performance of DMF (79%) and matching that of cyrene/EtOH (85%) (Scheme 10b). Importantly, the process mass intensity (PMI) was reduced from 114.2 in DMF to 17.9 in PC‐DMC, which is a direct consequence of the high substrate concentration achievable in PC‐DMC (5 mmol scale) enabled by the favourable solvation properties of PC and the reduced number of charge equivalents required (2.0 F vs. 6.2 F in DMF). The high dielectric constant of PC also contributed to improved process efficiency by allowing the salt loading to be reduced from 0.20 M under non‐optimised conditions to 0.15 M TBABF_4_ in the final protocol whilst still maintaining sufficient conductivity for effective charge transfer. For the anodic synthesis of diaryliodonium **48**, the PC‐DMC mixture was switched to afford a PC‐rich system (4:1 by volume) to maximise the polarity and conductivity for the demanding anodic oxidation (Scheme 10c). This delivered the iodonium salt in an 89% isolated yield, which is similar to the yields typically reported in the literature using MeCN (97%) [80] but crucially avoids the need for strong acids and fluorinated solvents.

Importantly, during the course of this study, some PC‐specific limitations emerged. First, under aerobic conditions in the cathodic ketone reduction (Scheme 10b), PC was found to participate as a reactant, generating a methylcarbonate by‐product **45** in a 32% yield. This reactivity is attributed to the inherent susceptibility of the cyclic carbonate moiety of PC to nucleophilic attack: the alkoxide intermediate generated upon the cathodic reduction of benzophenone attacks the electrophilic carbonyl carbon of PC, triggering ring opening and reductive transesterification. However, selectivity was restored by excluding O_2_ and adding a small amount of water. Second, in TEMPO oxidation, increasing the PC content beyond the optimised 1:4 ratio continuously reduced the catalytic rate due to viscosity‐limited TEMPO diffusion (Scheme 10a). Finally, the high boiling point of PC was explicitly addressed through three isolation strategies: liquid–liquid extraction with pentane, vacuum distillation and silica gel absorption. Whilst each was found to be effective at the laboratory scale, the authors acknowledged this would be challenging for upscaling, reinforcing the thermal persistence of PC as the most significant unresolved practical limitation across the synthetic transformations reviewed herein.

### 2.3 Asymmetric Hydrogenation Reactions

Biosca et al. [81] introduced PC in a different context from all the reactions previously discussed: asymmetric metal‐catalysed hydrogenation, where the solvent role is not to stabilise ionic intermediates or support electrolyte dissociation, but to dissolve the substrate and catalyst without interfering with the precise chiral environment required for enantioselective hydrogen delivery. The conventional solvent for these transformations is DCM. The authors demonstrated that PC could serve as a direct replacement in both Ir‐catalysed hydrogenation of minimally functionalised olefins and Rh/Ir‐catalysed hydrogenation of β‐enamides with no loss of enantioselectivity.

**SCHEME 11:**
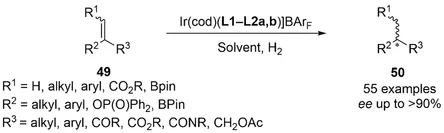
Ir‐catalysed asymmetric hydrogenation of minimally functionalised olefins.

**FIGURE 3:**
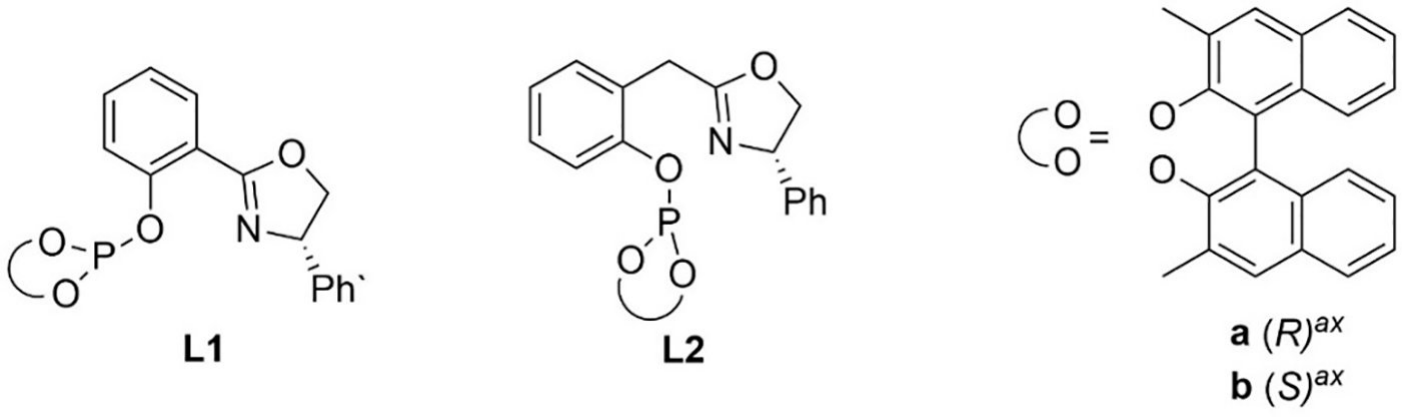
Chiral phosphite‐ozazoline ligands.

For the hydrogenation of trisubstituted olefins **50** (Scheme 11) using the best‐performing ligands **L1**–**L2** (Figure 3) under 100 bar H_2_ at room temperature with a 1 mol% Ir catalyst, full conversions of olefins **49** were achieved after 12 h with enantioselectivities essentially identical to those in DCM, as shown in Table 6. As noted, a higher H_2_ pressure was required in PC (100 bar) compared to DCM (50 bar) for the olefin hydrogenation, and longer reaction times were needed (12 vs. 2 h), which suggests that hydrogen solubility or mass transfer is less favourable in PC than in DCM, which is a direct consequence of PC’s higher viscosity (*η* = 2.53 mPa s) and density compared to DCM (*η* = 0.38 mPa s). This represents a practical limitation, as higher pressures require specialised equipment and increase safety and operational demands.

**TABLE 6:** Ir‐catalysed hydrogenation of selected example substrates (**50a–c**) using ligands **L1** and **L2**.

			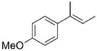 % ee^c^	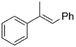 % ee^c^	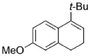 % ee^c^
Entry	L	Solvent	50a	50b	50c
1^a^	**L1b**	DCM	15	19	89
2^a^	**L2a**	DCM	94	96	48
3^a^	**L2b**	DCM	95	97	51
4^b^	**L1b**	PC	13	21	89
5^b^	**L2a**	PC	93	96	46
6^b^	**L2b**	PC	95	96	54

a Reactions carried out at room temperature by using 0.5 mmol of substrate and 1 mol% of Ir‐catalyst precursor at 50 bar of H_2_ using DCM (2 mL) as solvent. Full conversions were achieved after 2 h.

b Reactions carried out using PC (2 mL) as solvent and 1 mol% of Ir‐catalyst precursor at 100 bar of H_2_. Full conversions were achieved after 12 h.

c Enantiomeric excesses measured by chiral GC or HPLC.

**SCHEME 12:**
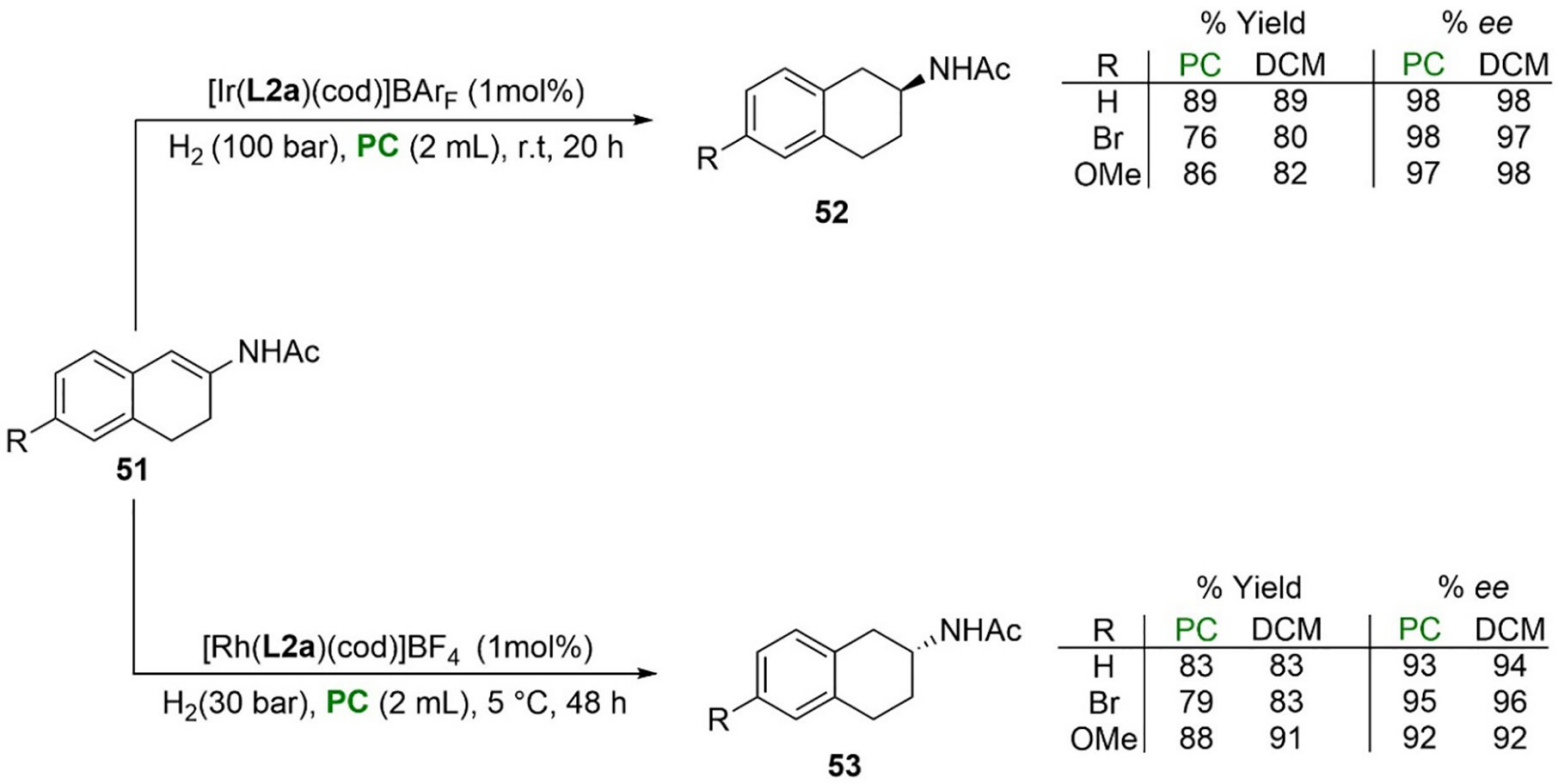
Asymmetric hydrogenation of cyclic β‐enamides **51** in PC.

On the other hand, for the reduction of various β‐enamides **51** using the best performing ligand **L2a**, an Ir **61**  catalyst in PC (100 bar H_2_) delivered tetrahydronaphthalenes **52** in yields of 76%–89% and 97%–98% ee, whilst switching to a Rh catalyst (30 bar H_2_) afforded the enantiomeric tetrahydronaphthalenes **53** in 79%–88% yields and 92%–95% ee, all comparable to the results in DCM (Scheme 12).

However, it is noteworthy that the Rh‐catalysed reactions in PC required significantly longer reaction times than those in DCM (48 vs. 20 h), again consistent with viscosity‐limited hydrogen diffusion. Despite these limitations, the authors highlight the compatibility of PC with the catalytic system of this transformation as a key advantage, noting that it makes the new ligands particularly appealing for industrial applications and enhances their sustainability.

**FIGURE 4:**
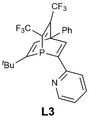
Structure of the 1‐phosphabarrelene‐pyridine ligand **L3**.

A later investigation by Biosca and co‐workers [82] demonstrated a similar Ir‐catalysed reduction of aminonaphthalenes **54** that utilised a structurally novel ligand class (chiral 1‐phosphabarrelene‐pyridine P, N ligands, **L3**, Figure 4) and further demonstrated the suitability of PC towards asymmetric hydrogenation reactions. PC afforded analogous results to the traditional solvent, DCM, with full conversion and slightly improved enantioselectivity (95% vs. 93% ee in DCM) (Table 7).

**TABLE 7:** Ir‐catalysed asymmetric hydrogenation of cyclic β‐enamides.^a^

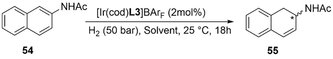			
Entry	Solvent	% Conv (% yield)^b^	% ee^c^
1	DCM	100 (96)	93
2	PC	100	95

a Reactions carried out at 0.5 mmol scale using 2 mL of solvent.

b Conversions measured by GC. Isolated yields in parentheses.

c Enantiomeric excesses measured by GC or HPLC.

The authors also reported a Rh‐catalysed hydrogenation of β‐dehydroamino acid derivatives **56** (5 bar H_2_, 5 °C) that was originally performed in an alternative traditional solvent, THF, but which performed equally well in PC, plus another green solvent, 2‐MeTHF (95% ee in all solvents) (Table 8). Across all these examples, it has been demonstrated that PC can successfully substitute halogenated and other polar aprotic traditional solvents in sensitive asymmetric transformations without compromising the stereochemical integrity of the product.

**TABLE 8:** Rh‐catalysed asymmetric hydrogenation of β‐dehydroamino acids.^a^

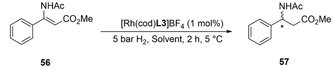			
Entry	Solvent	% Conv (% yield)^b^	% ee^c^
1	THF	100 (95)	95
2	2‐MeTHF	100	95
3	PC	100	95

a Reaction carried out at 0.5 mmol scale using 2 mL of solvent.

b Conversions measured by GC. Isolated yields in parentheses.

c Enantiomeric excesses measured by GC or HPLC.

### 2.4 CO_2_‐Based Reactions (Carboxylation and Cycloaddition)

**SCHEME 13:**
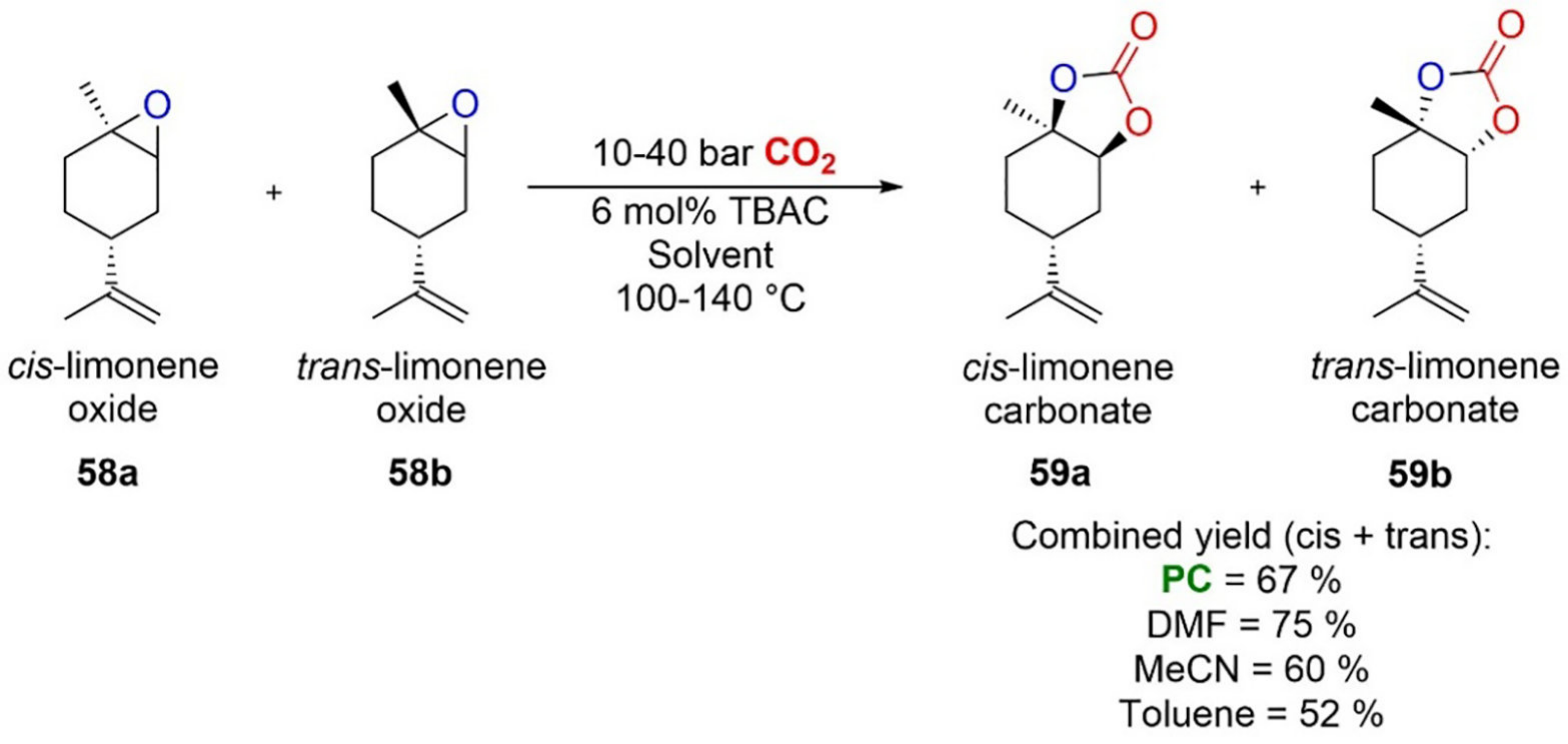
Cyclic carbonate synthesis via CO_2_ cycloaddition.

The cycloaddition of CO_2_ to epoxides to produce five‐membered cyclic carbonates is a 100% atom‐economic transformation of growing interest in green chemistry (see the methods of PC synthesis in Section 1). In this context, Rehman et al. [83] reported an evaluation of PC alongside DMF, MeCN and toluene for the synthesis of limonene carbonate (LC) **58** from bio‐based limonene oxide (LO) **59** and catalysed by tetrabutylammonium chloride (TBAC) (Scheme 13). The solvent performance was assessed through the pseudo‐first‐order observed rate constant (*k*_obs_), which, under the semi‐batch conditions of this study, where CO_2_ is present in large excess, and TBAC concentration remains constant, captures the net effect of the solvent environment on the overall reaction rate; therefore, a higher *k*_obs_ directly reflects a more kinetically favourable medium.

The solvent screening demonstrated that PC performed second‐best (*k*_obs_ = 0.1312 h^−1^) behind DMF (0.1769 h^−1^) and ahead of MeCN (0.1157 h^−1^) and toluene (0.0753 h^−1^). The authors attributed the performance of PC to its high dielectric constant and polarity, which allow it to effectively solubilise CO_2_ and the nucleophilic halide cation (Cl^−^), as well as stabilise ionic intermediates during epoxide ring opening. The solvation capacity of PC helped to maintain homogeneous conditions and avoid deleterious catalytic activity, properties that were essential to obtain a reliable mechanistic and kinetic analyses. The high boiling point of PC also ensured thermal stability across the full temperature range investigated (100–140 °C).

**FIGURE 5:**
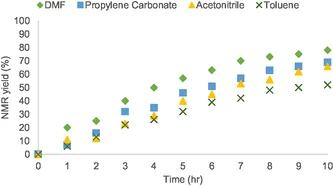
Plot of NMR spectroscopic yield of LC **59** versus reaction time in different solvents.

Although PC achieved good yields compared to MeCN and toluene (Figure 5), in this case, the superior performance of DMF was attributed to its additional role as a nucleophilic activator of CO_2_ through the amide group, a synergistic catalytic contribution that PC does not replicate, despite the structural similarity between amides and carbonates. Whilst PC was chosen as the solvent for the reaction, given its green credentials, the structural similarities between the solvent and product could potentially lead to issues with isolation and purification, although these were not raised in the report.

In a subsequent study by the same group [84], the CO_2_ cycloaddition of styrene oxide (SO) **61** was investigated using a binary ZnBr_2_/TBAB catalyst system, with PC again selected as the reaction solvent for the formal kinetic study. The initial solvent screening revealed a markedly different ranking from that observed in the limonene oxide (**59**) system, as MeCN outperforms all other solvents (Table 9). In terms of catalytic efficiency, PC achieves a turnover frequency (TOF) of 487.5 h^−1^, which is lower than that of MeCN but higher than that of toluene and DMF, confirming that it represents a reasonable compromise between performance and sustainability. Although the authors do not discuss the inversion of the relative performance of DMF and PC, compared to the previous study, these results may reflect the different catalytic system: the Lewis acidic ZnBr_2_ could bind unfavourably with the amide group of DMF, partially deactivating the catalyst, with a lesser effect expected for the carbonate in PC.

**TABLE 9:** Effect of solvent on styrene carbonate (SC) formation catalysed by ZnBr_2_/TBAB.^a^

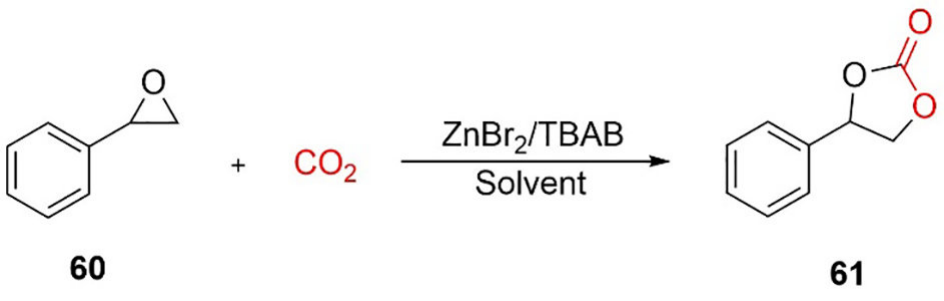			
Entry	Solvent	Conversion, %	TOF, h^−1^ b
1	Toluene	67	466.7
2	DMF	55	394.6
3	MeCN	81	550.4
4	PC	73	487.5

a Reaction conditions: 4.5 M styrene oxide in solvent, TBAB (0.1 M), ZnBr_2_ (6.5 mM) at 100 °C and 6 bar CO_2_.

b Turnover frequency (TOF) = (moles of product)/(moles of catalyst·time).

Beyond the reaction performance, this paper reveals a practically significant analytical limitation; the carbonyl absorption at 1800 cm^−1^ in the solvent directly overlaps with the cyclic carbonate stretch in the product (**61**)**,** rendering impossible the direct FTIR monitoring of product formation. This spectral overlap is an inherent consequence of the cyclic carbonate structure of PC and represents a recurrent analytical limitation in this class of reaction, in addition to the potential separation issues noted in the limonene carbonate study.

**SCHEME 14:**
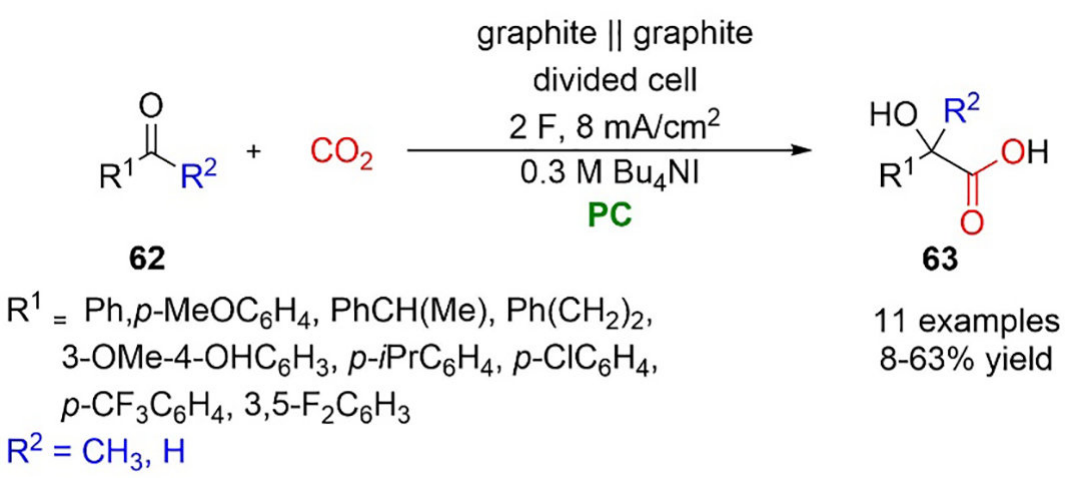
Electrocarboxylation reaction in PC at graphite cathodes.

In a mechanistically distinct application of PC and CO_2_ utilisation, Seidler and collaborators reported the electrocarboxylation of aldehydes and ketones to yield α‐hydroxy acids **63** (Scheme 14) [85]. PC outperformed all alternative solvents tested (MeCN, DMF and DMSO), delivering mandelic acid in 27% yield via bulk electrolysis versus <15% in the other solvents. Following optimisation of reaction conditions, PC afforded yields up to 63% for mandelic acid from benzaldehyde (**62**, R^1^ = Ph, R^2^ = H). This is an interesting finding given the lower CO_2_ solubility in PC (134 mmol/L) compared to that in MeCN (314 mmol/L) and DMF (194 mmol/L). The authors emphasised that despite the similar CO_2_ solubilities of PC and DMSO, they delivered different yields and that this discrepancy suggests that CO_2_ solubility alone does not govern the carboxylation efficiency in this system.

The authors attribute the superior performance of PC to a combination of its high dielectric constant, low residual water content after drying (136 ppm)—compared to MeCN (168 ppm), DMSO (137 ppm) and DMF (305 ppm)—which suppresses competing alcohol and pinacol‐type side products, and an exceptionally wide electrochemical stability window that allows reactions to be conducted at different potentials without the risk of solvent decomposition.

### 2.5 Peptide Synthesis

**SCHEME 15:**
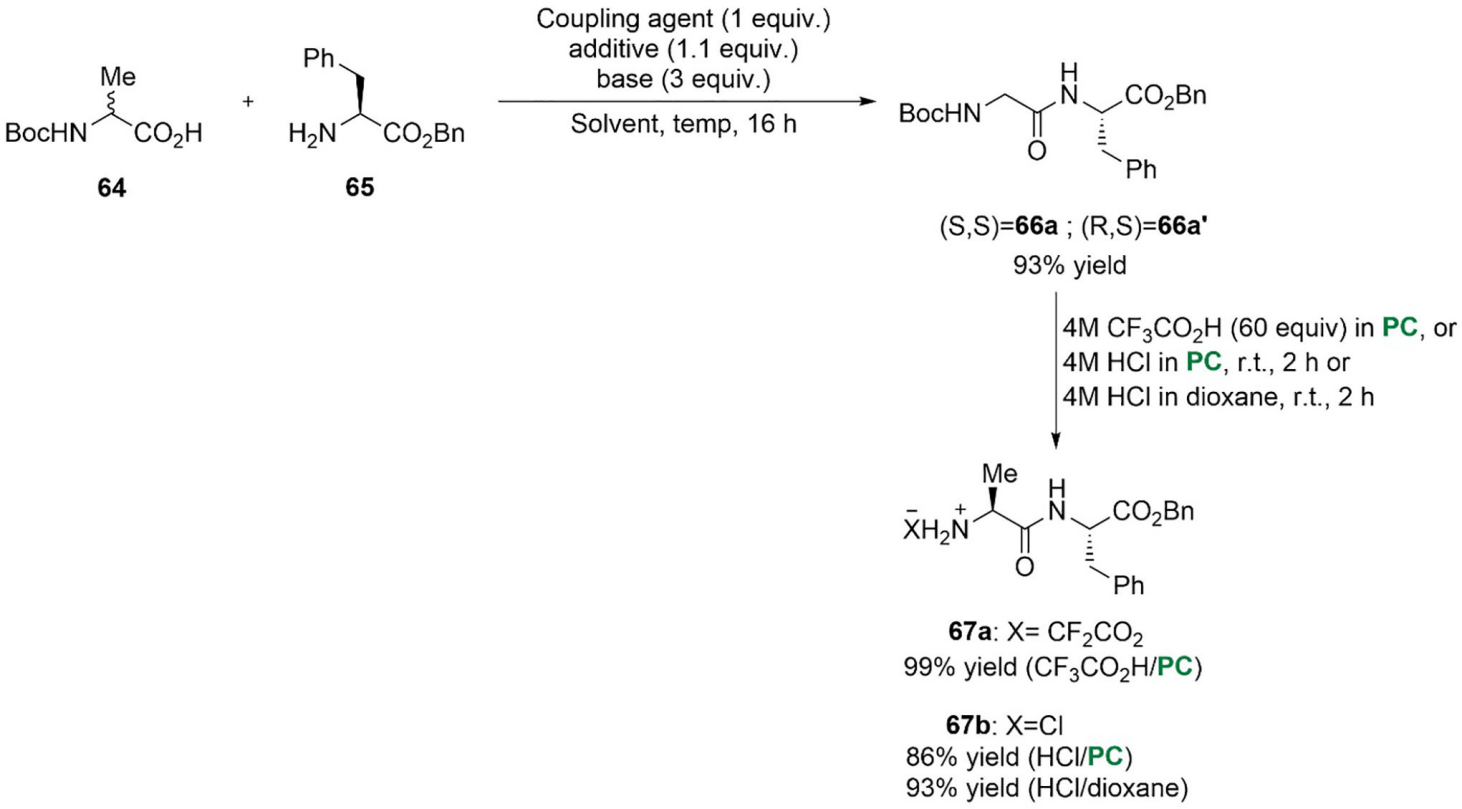
Synthesis of dipeptides **66–67** in PC.

Lawrenson et al. reported the use of PC as a green solvent replacement for DMF and DCM in both solution‐ and solid‐phase peptide synthesis [86]. In solution‐phase synthesis, PC exceeded the performance of the conventional solvents under optimised conditions (EDC/HOBt, *i*Pr_2_EtN, 20 °C), achieving the desired dipeptide **66a** in a 93% yield, compared to 81% in DCM/DMF and 83% in DMF alone (Table 10). The authors attributed this superior performance primarily to the differing dielectric constant and dipole moment, both exceeding those of DMF and DCM, which favours the stabilisation of the charged transition states involved in amide bond formation. Further evaluation of the versatility of PC was performed through the synthesis of various tetrapeptides (80%–99% yields) (Scheme 15). Subsequent *N*‐Boc deprotection with trifluoroacetic acid proceeded in a 99% yield in PC, surpassing the 93% obtained with 4 M HCl in dioxane.

**TABLE 10:** Synthesis of dipeptide **66a**.^a^

Entry	*T*, °C	Solvent	Yield
1	20	PC	93
2	20	CH_2_Cl_2_:DMF	81
3	20	DMF	83
4	70	DMF	78
5	70	PC	82

a EDC (*N*‐ethyl‐*N*‐(3‐dimethylaminopropyl)carbodiimide) and HOBt (hydroxybenzotriazole) as coupling agent and additive. *N–N‐*Diisopropylethylamine as base.

**FIGURE 6:**
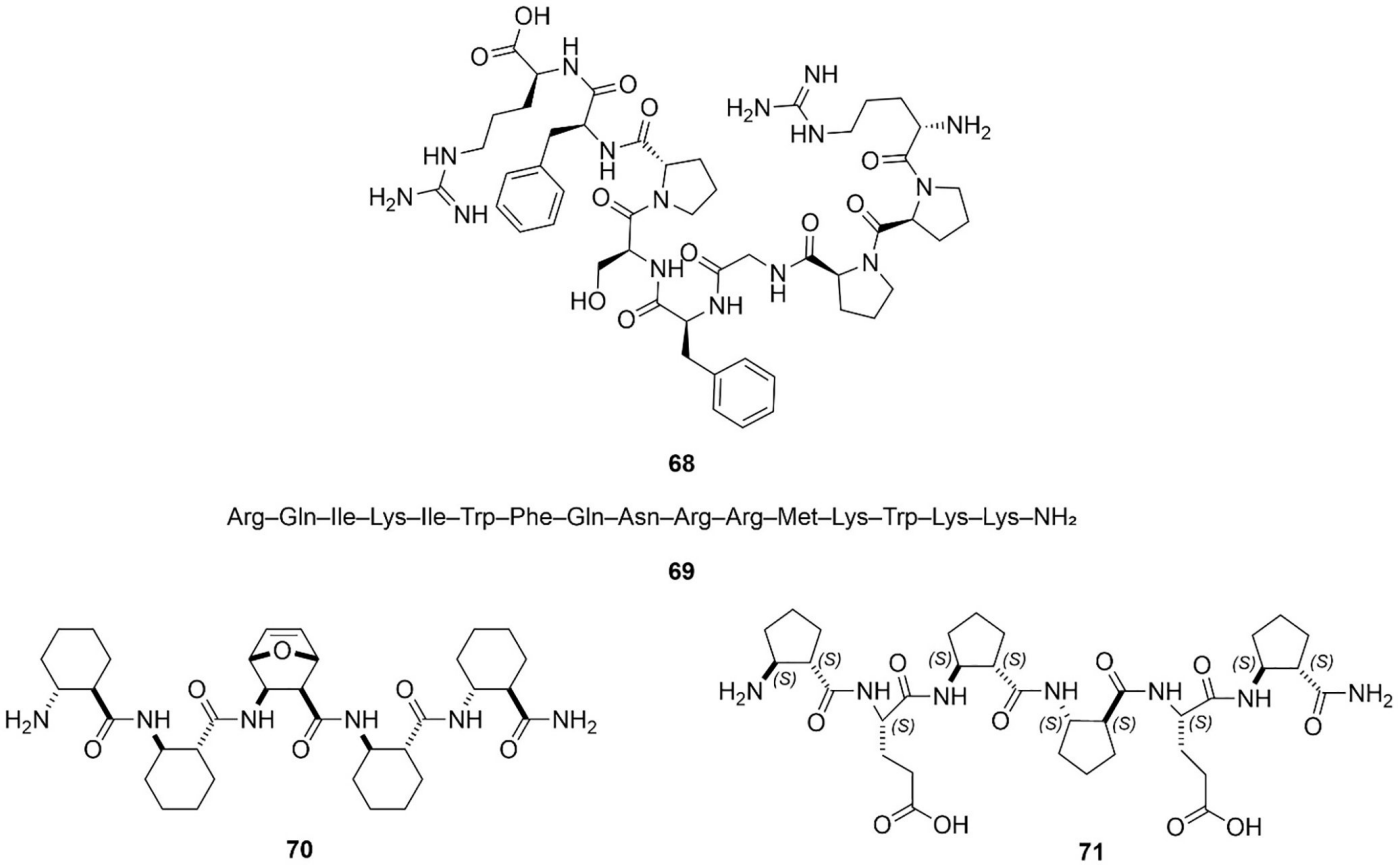
α‐ and β‐peptides synthesised using PC as solvent.

The synthesis of the biologically relevant nonapeptide bradykinin (H‐Arg‐Pro–Pro‐Gly‐Phe‐Ser‐Pro‐Phe‐Arg‐OH, **68**, Figure 6) in PC afforded a purity of 77%, comparable to the 79% obtained in DMF and notably with a more favourable impurity profile, as the premature chain termination sequences present as overlapping impurities in the DMF sample were absent in the PC one, facilitating its purification. However, the high boiling of PC meant that the residual solvent needed to be removed by short‐path distillation, which, whilst feasible, adds an extra step compared with other solvents (e.g. DCM); a recurrent issue within this review. It should be noted that PC does not adequately swell polystyrene‐based resins, restricting its use to polyethylene glycol‐based supports such as the ChemMatrix resin; however, as the results above demonstrate, this does not necessarily translate into a practical limitation, and the compatibility of PC with the ChemMatrix resin proved sufficient to deliver outcomes comparable to those obtained with DMF. This swelling behaviour of PC with other resin types is nonetheless a factor worth considering when extending its use to a broader range of solid‐phase synthesis applications.

Building directly on the batch solid‐phase peptide synthesis work of Lawrenson et al., Varro and collaborators extended the use of PC to continuous‐flow solid‐phase peptide synthesis (CF‐SPPS) [87]. In addition to demonstrating the compatibility of PC with a technologically more demanding format, this report addresses two limitations of conventional SPPS: the use of hazardous solvents and process inefficiency. Four α‐peptides were synthesised with raw purities above 95% and yields of 91%–96%. The methodology was further validated on structurally challenging β‐peptides (**69–71**, Figure 6), with comparable performance (>96% raw purity and >89% yield).

The most distinctive contribution of this paper is the quantitative demonstration that the CF‐SPPS process using PC reduces solvent consumption by approximately two orders of magnitude compared to classical SPPS (Table 11), and PMI values drop from above 50,000 in classical SPPS to between 564 and 681 in CF‐SPPS. It is noteworthy that a limitation specific to the HATU/DIPEA combination of coupling reagents was discovered in PC, as precipitation blocked the reactor tubing, so an alternative set of coupling reagents (OxymaPure/DIC) was required for β‐amino acid couplings. As this precipitation does not occur in DMF, the authors recommend evaluating the reagent system on a case‐by‐case basis in PC.

**TABLE 11:** Selected examples of the synthesised α‐ and β‐peptides in PC as solvent.

Compound	Time, min		Solvent used, mL		PMI value		Raw purity, %	Yield, %
	Classic SPPS	CF‐SPPS	Classic SPPS	CF‐SPPS	Classic SPPS	CF‐SPPS		
**69**	~2580	~381	~5310	~57.2	51,062	564	>97	93
**70**	~1120	~357	~1680	~18.6	57,895	659	>96	89
**71**	~1100	~240	~2010	~22.1	60,302	681	>98	93

### 2.6 Addition Reactions and C─C/C─O Bond Formation

Yang et al. reported the phosphomolybdic acid (H_3_PMo_12_O_40_, POM) catalysed carbohydroxylation of terminal alkynes with benzylic alcohols to yield β‐arylethyl ketones, a C─C bond‐forming reaction where PC proved superior among all solvents tested (Table 12) [88]. For example, protic solvents such as ethanol, water and DMF gave negligible yields (<2%), whilst non‐polar and halogenated solvents afforded moderate yields (46%–64% yield) but with unsatisfactory levels of product selectivity. Notably, both organic carbonate solvents out‐performed DCM (59% yield), with PC (99%) superior to ethyl carbonate (76%). Supported by an Electrospray Ionisation Mass‐Spectrometry (ESI‐MS) analysis, the identification of an adduct between PC and the phosphomolybdic acid, with formula [(HPMo_12_O_40_) + 2 PC + H_2_O]^2−^ suggest that PC associates with the catalyst and may stabilise the heteropolyanion, whilst also potentially solvating the carbocation intermediate generated from the benzyl alcohol, pointing to a dual role for PC rather than a passive solvent.

**TABLE 12:** Substrate scope of H_3_PMo_12_O_40_‐catalysed carbohydroxylation reaction in PC.

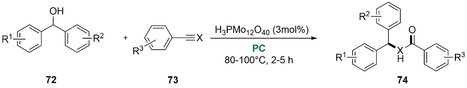				
Entry	Alcohol	Alkyne	Conditions	Yield, %
1	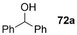	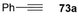	80 °C, 2 h	91
2	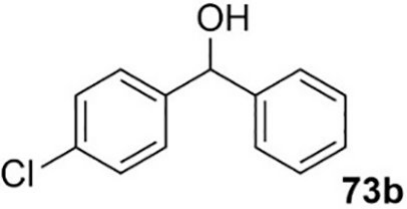	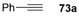	80 °C, 2 h	93
3	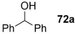	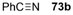	100 °C, 5 h	88
4	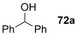	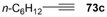	100 °C, 4 h	35
5	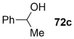	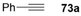	80 °C, 3 h	56
6	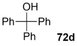	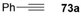	80 °C, 3 h	14

An investigation into the substrate scope revealed limits to this stabilising effect. Whilst the system performs well with secondary diaryl methanols and aromatic alkynes (Table 12 entries 1 and 2, 91% and 93% yields, respectively) as well as nitriles via a Ritter‐type reaction (entry 3, 88% yield), it is less effective for aliphatic alkynes (entry 4, ~35%, but product not isolable), singularly benzylic secondary alcohols (entry 5, 56%) or tertiary alcohols (entry 6, 14%), even at more elevated temperatures (100 °C). In this context, the inability to readily generate a stable carbocationic intermediate confirms that PC’s stabilising capacity is selective, and it must be evaluated on a case‐by‐case basis. Furthermore, regarding isolation protocols, the process includes an aqueous workup, ether extraction, and column chromatography regardless of the solvent used, and notably, the authors did not address whether the high boiling point further complicated product recovery, a significant challenge frequently observed in the literature previously discussed.

**SCHEME 16:**
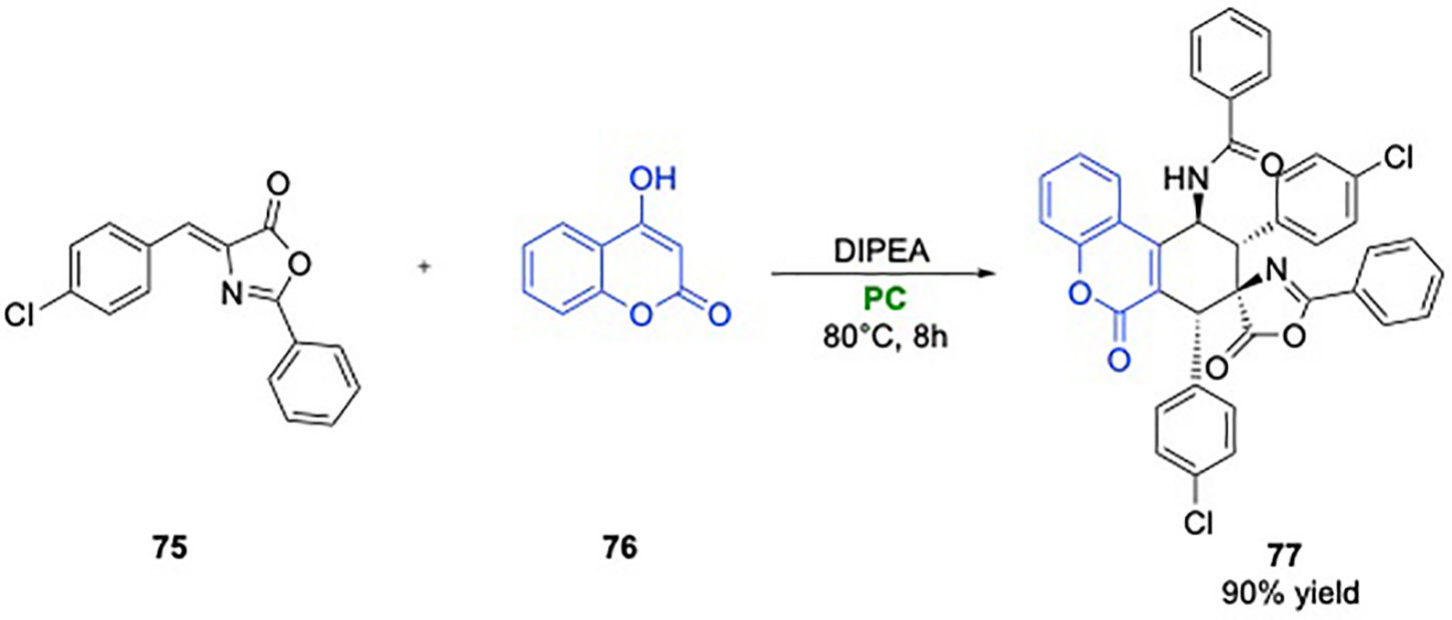
Synthesis of tetrahydro‐6*H*‐spiro[benzo[c]chromen‐6‐one]oxazolone in PC.

Expanding the application of PC beyond C─C bond formation, Shafiee et al. reported a three‐component cascade reaction of arylidene azlactones **75** and 4‐hydroxycoumarins **76** to yield dihydro‐5′*H*‐spiro[benzo[c]chromene‐8,4′‐oxazole]‐5′,6(7*H*)‐diones **77** as unusual amino acid scaffolds [89]. The solvent screening revealed a clear performance trend, with PC delivering 90% yield of the model compound tetrahydro‐6H‐spiro[benzo[c]chromen‐6‐one]oxazolone **77** (Scheme 16), out‐performing toluene (70%), MeCN (50%), DMSO (20%) and DMF (17%), whilst PEG‐200 (polyethylene glycol 200) gave no product at all. The superior performance of PC is consistent with its high polarity and aprotic character, which facilitates the ionic intermediates involved in the cascade mechanism whilst avoiding the protic interference that would disrupt the reaction pathway. However, the authors note one chemical limitation to the use of PC in this transformation, as during optimisation, PC was found to be unstable in the presence of strong acids or bases, releasing CO_2_, thereby necessitating the use of DIPEA as a mild base.

Unlike several of the reaction systems previously discussed in this review, where the high boiling point of PC complicated product isolation, this study presents a notably cleaner procedure, where product isolation did not require organic extraction or chromatography. Instead, the product precipitated upon addition of water and was recovered by simple filtration, after which PC was recovered by evaporation of the aqueous phase and directly reused without further purification. The crude product was subsequently purified by recrystallisation from hot ethanol, which is a straightforward and low‐waste alternative to column chromatography. PC was successfully recycled up to five times, with only a small decline in yields (from 90% to 80%), which confirms its chemical stability under these mild base conditions. Furthermore, the authors successfully achieved the scale‐up synthesis of compound **77** at five times the standard scale without modification of the protocol, delivering 2.67 g in an 88% yield and with 94% atom economy.

**FIGURE 7:**
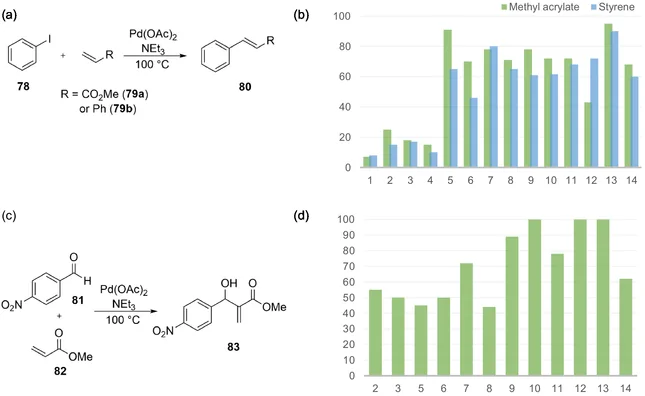
(a) Scheme of the Heck reaction. (b) Conversion (%) of the Heck reaction after 24 h. (c) Scheme of the Baylis–Hillman reaction. (d) Conversion (%) of the Baylis–Hillman reaction. The numbers 1–14 correspond to the solvents in Table 13.

A comparative study by Sangon et al. provides a broader evaluation of PC, benchmarking it directly with 14 other solvents (Table 13), including emerging green alternatives (NBP, GVL, Cyrene), across two pharmaceutical‐relevant C─C bond‐forming reactions: the Heck reaction and the Baylis–Hillman reaction [90]. The study analysed the the Linear Solvation Energy Relationship (LSER) across all solvents in both reactions and confirmed solvent dipolarity as the determining factor in reactivity. For example, in the Heck reaction (Figure 7a,b), PC delivered moderate conversion after 24 h, less than the highest‐performing solvents, sulfolane (~95%) and NBP (~90%), and consistent with its intermediate position on the dipolarity scale. Beyond the stabilisation of polarised transition states, it was proposed that solvent dipolarity plays a broader role in supporting catalyst activity throughout the reaction: higher‐polarity media favour the generation and stabilisation of the active palladium species whilst delaying their decomposition, resulting in the superior conversions observed in these solvents.

**TABLE 13:** Selected solvents used in the study.

	Solvent	Dipolarity (π*)
1	Limonene	0.24
2	P‐Cymene	0.39
3	CPME	0.42
4	Toluene	0.50
5	NBP	0.77
6	MeCN	0.80
7	GVL	0.83
8	DMAc	0.85
9	DMF	0.88
10	NMP	0.90
11	PC	0.90
12	Cyrene	0.93
13	Sulfolane	0.96
14	DMSO	1

On the other hand, in the Baylis–Hillman reaction (Figure 7c,d), PC performed notably better, out‐performing DMF and NMP across an extended substrate scope and achieving competitive conversions with sulfolane and DMSO. This was attributed to the effective stabilisation of the charged intermediates generated along the catalytic cycle, which, for this transformation, are particularly sensitive to solvent dipolarity.

However, a limitation of this study is that reactions in PC yield crude product mixtures inflated (>100%) by residual solvent contamination, requiring additional purification by column chromatography. In contrast, when the reaction was performed in an alternative green solvent, Cyrene, the product could be isolated by simple aqueous extraction. This result highlights the frequent work‐up limitation with PC and demonstrates that its high boiling point may be a practical obstacle to the uptake of PC solvent usage, despite its otherwise favourable sustainability profile.

**SCHEME 17:**
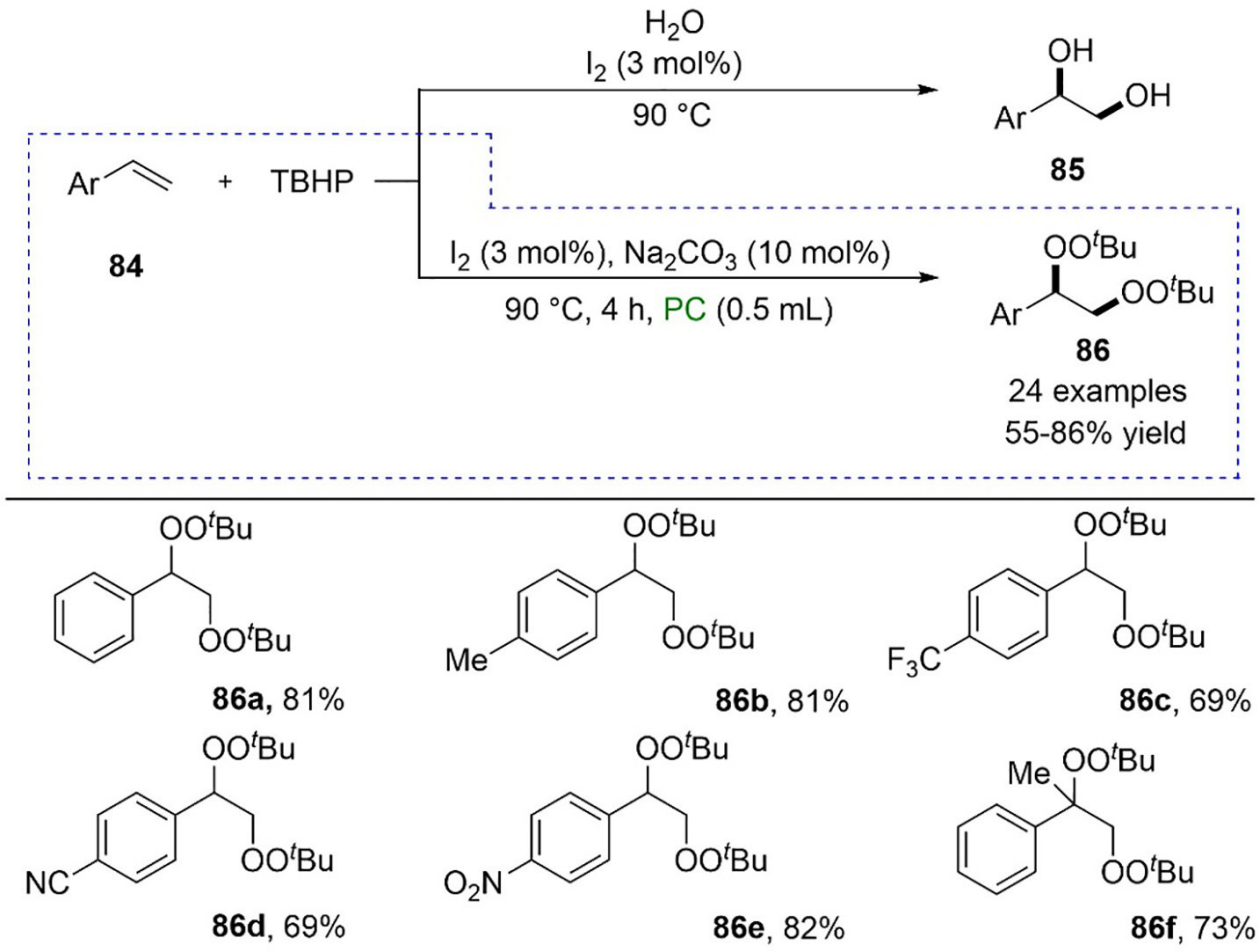
Approaches to dioxygenation of alkenes in H_2_O and PC, and selected examples of the synthesis of bisperoxides in PC.

Gao et al. reported the iodine‐initiated dioxygenation of styrenes **84** using *tert*‐butylhydroperoxide (TBHP) as both oxidant and oxygen source, a metal‐free protocol that selectively produces either vicinal diols **85** or vicinal bisperoxides **86** depending on the solvent and additives employed (Scheme 17) [91]. PC plays a fundamentally different role in this transformation, as it is specifically selected not only for its green profile and high levels of conversion but also as the optimal solvent for the bisperoxides pathway. Whilst water selectively promotes vicinal diol formation via epoxide ring opening, PC directs the reaction towards vicinal bisperoxides (**86**) by suppressing the hydrolysis pathway and favouring radical combination of the benzylic intermediate with a second ^*t*^BuOO radical. Under optimal conditions, PC delivered the model substrate **86a** in 82% yield, out‐performing MeCN (51%) and solvent‐free conditions (55%), whilst water produced a mixture of diol and bisperoxide (18% diol **85** + 61% bisperoxide **86**). The solvent screening also demonstrates that reducing the amount of either TBHP or PC led to dramatic reductions in yield (38%). This indicates that the concentration in PC is important, with the solvent likely providing the medium required to dissolve and concentrate the radical species and suppress competing water‐mediated pathways. The authors explicitly attribute this selectivity to the non‐nucleophilic nature of PC, rendering it unable to promote competing epoxide ring opening.

A broad substrate scope was reported in PC, tolerating electron‐donating (4‐Me) and electron‐withdrawing (4‐CF_3_, 4‐CN, 4‐NO_2_) aryl substituents, as well as α‐substituted styrenes (**86f**, 73% yield). However, the lack of reactivity of aliphatic alkenes, such as cyclohexene, restricts the scope to aryl‐conjugated systems where benzylic radical stabilisation is available. Whilst the adoption of PC and TBHP improves the green profile of this transformation, the downstream processing remains complex, as the isolation of the product requires decolourisation with Na_2_S_2_O_3_, aqueous washing, multiple ethyl acetate extractions and purification via column chromatography using a highly apolar eluent system (petroleum ether/EtOAc ratios up to 400:1). Whilst the bisperoxide products 86 are sufficiently apolar to be separable from PC, the overall process remains solvent‐intensive (note: the authors do not mention whether PC is recovered after the wash step).

### 2.7 Substitution, Alkylation and Condensation Reactions

**SCHEME 18:**
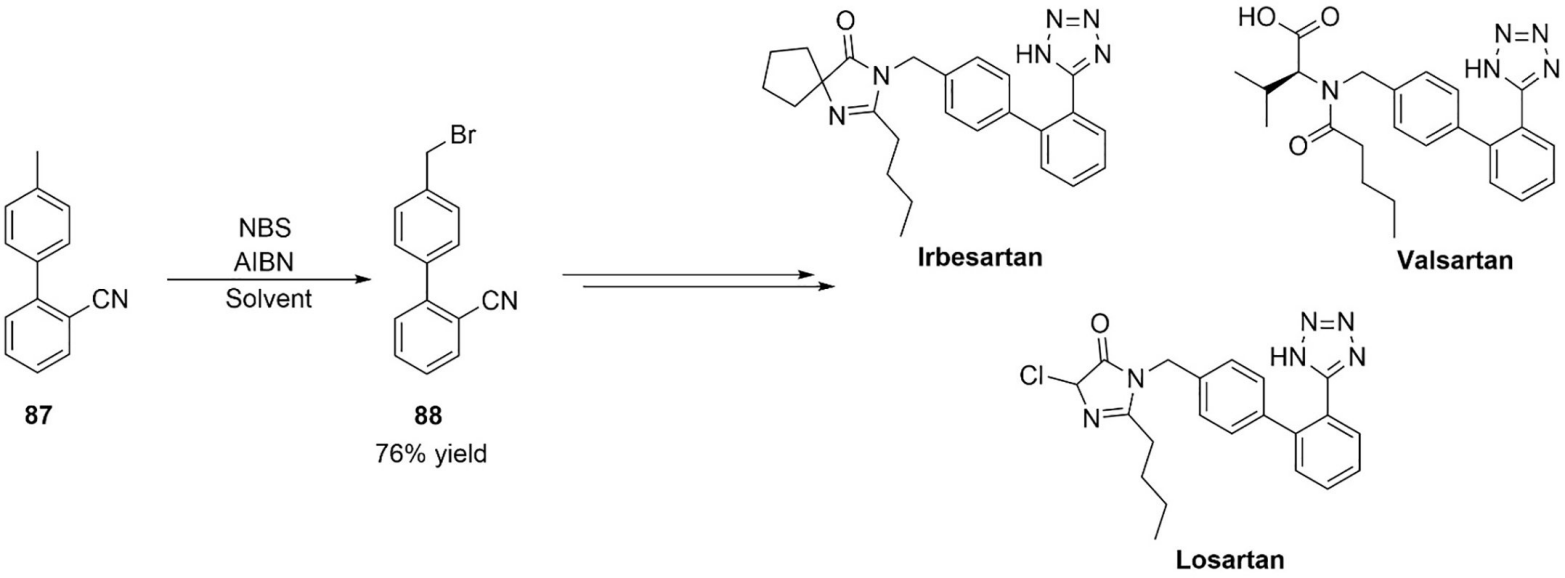
Bromination of 2‐cyano‐4′‐methylbiphenyl (**87**) to afford 4′‐(bromomethyl)‐2‐cyanobiphenyl (**88**) using *N‐*bromosuccinimide (NBS).

In a pharmaceutical synthesis context, Sveegaard and Kristiansen reported a multivariate solvent selection study for the Wohl‐Ziegler bromination of 2‐cyano‐4′‐methylbiphenyl (**87**) to afford 4′(bromomethyl)‐2‐cyanobiphenyl (BCB) **88** (Scheme 18) [92]. This is a key building block in the industrial synthesis of sartans, a widely prescribed class of antihypertensive drugs, whose traditional synthesis uses toxic solvents such as chlorobenzene, dichloroethane or carbon tetrachloride [93, 94]. To systematically map the solvent space and predict reaction performance, the authors employed Response Surface Methodology (RSM) integrated with Principal Component Analysis (PCA) of solvent physicochemical properties. Unlike conventional one‐variable‐at‐a‐time screening, this approach reveals complex interaction effects between solvent properties. The PCA scores condense individual parameters into two main components: *t*_1_ (polarity and solvation capacity) and *t*_2_ (hydrogen‐bonding donor/acceptor ability). The model identifies the high‐performance region as *t*_1_ ≤ −2 and *t*_2_ ≤ −3 for maximum yield (≥80%), with the optimal point within the model domain at *t*_1_ = −3.04, *t*_2_ = −3.34, corresponding to nitromethane, which was the solvent that delivered the highest experimental yield (80%) and conversion (90.9%) in the study. PC, with scores of *t*_1_ = −3.56 and *t*_2_ = − 0.74, approaches this region along *t*_1_ but falls short along *t*_2_, which explains why its performance is good but not optimal: PC delivered 76% yield and 84% conversion, matching chlorobenzene (73% yield) and slightly lower than DMC (79% yield), 1‐nitropropane (78%) and acetone (84.8%), as shown in Table 14.

**TABLE 14:** Solvent screening for the conversion of **87** into **88** in 9 different solvents.^a^

Solvent	*t*_1_	*t*_2_	Conversion of 87, %	Yield of 88, %
*n‐*Butyl acetate	1.58	0.12	85	72
1,3‐Dichlorobenzene	4.74	−1.68	87	72
Ethyl acetate^b^	−0.65	−1.5	88	77
Fluorobenzene	2.4	−4.14	89	75
*n*‐Hexyl acetate	3.31	1.97	80	68
Nitromethane	−3.84	−4.71	91	80
1‐Nitropropane	−0.64	−2.65	89	78
Propylene carbonate	−3.56	−0.74	84	76
Dimethyl carbonate (DMC)	−0.57	−2.59	89.7	79

a Reaction conditions: 1.55 mmol of **87**, NBS (1.05 equiv.), AIBN (0.0775 mmol), 2 mL of solvent. 80 °C, 3 h.

b Reaction time 6 h.

The authors attribute the success of the carbonate solvent class to its unique combination of moderate to low nucleophilicity, in addition to inertness towards NBS and benzyl bromide intermediates. This is critical, as it prevents deleterious side reactions that typically disqualify other polar media such as DMF and NMP (6% and 0% yield, respectively), suggesting that polarity alone is insufficient for success and that solvent‐reagent compatibility is a critical constraint in radical bromination. However, no data are provided regarding product isolation from PC, solvent recovery, or the overall work‐up procedure. In this context, whilst PC is an efficient reaction medium for this transformation, practicality in downstream processing remains unassessed.

The scope of pharmaceutical synthesis was further extended by Litim et al., who reported a catalyst‐free condensation of sulphonamides **89** with aldehydes **90** for the synthesis of *N‐*sulfonylimines **91** mediated by neutral Al_2_O_3_ as a reusable dehydrating agent [95]. The reaction is thermodynamically challenging because the imine product is prone to hydrolysis, without an effective water scavenger, thus limiting conversion. A solvent screening revealed that PC delivered 96% sulphonamide conversion and was superior to other traditional solvents such as ethyl acetate (47%), toluene (61%) and DCM (39%), whilst ethanol gave no conversion at all (Table 15). DMC promoted full conversion (100%), and the authors attribute the superior performance of the cyclic carbonates to their high dielectric constant, good solvation ability and polar aprotic character, which, as observed throughout this review, stabilise both the ionic transition states involved in the imine formation and the Al_2_O_3_ surface interactions that facilitate water adsorption.

**TABLE 15:** Synthesis of *N*‐sulfonylimine **91** from sulphonamides **89** in different solvents.

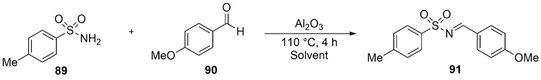		
Entry	Solvent	Conversion, % 91
1	DMC	100
2	PC	96
3	Toluene	61
4	AcOEt	47
5	DCM	39

**SCHEME 19:**
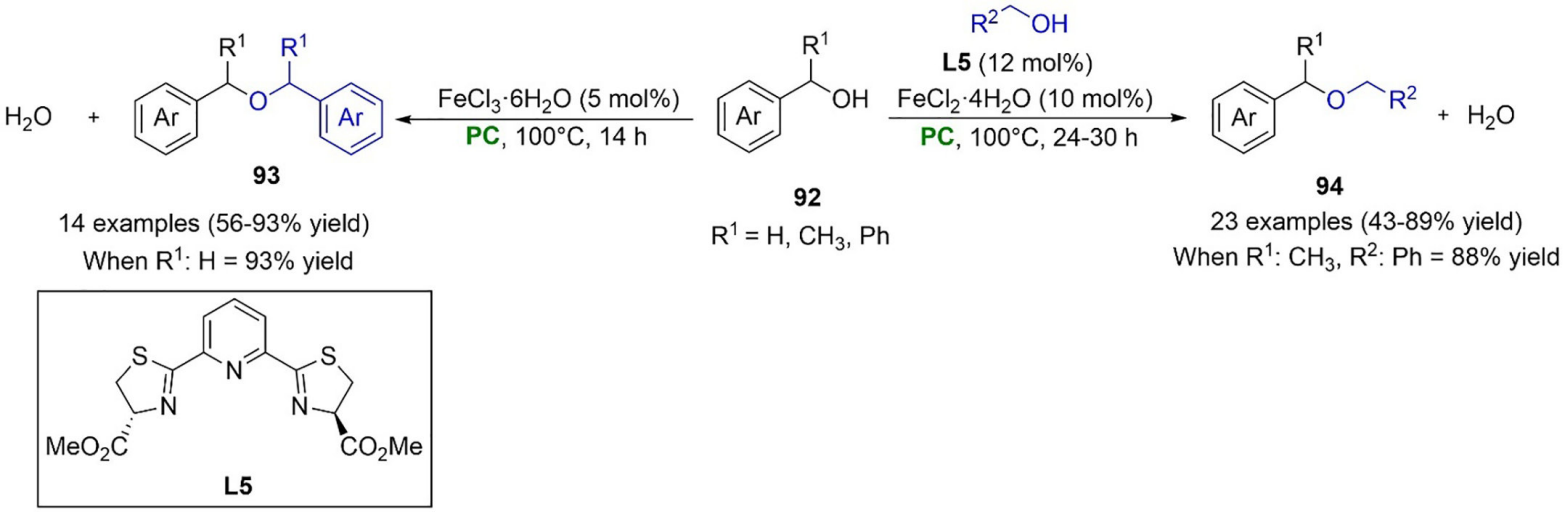
Homo‐ and cross‐etherification of alcohols.

In a follow‐up study from the same research group, Litim and co‐workers extended the application of PC to iron‐catalysed homo‐ and cross‐etherification of benzyl alcohols **92**; a C─O bond‐forming reaction that produces only water as a by‐product and represents a direct and atom‐economical alternative to the Williamson method (Scheme 19) [96]. Solvent screening confirms PC as the optimal medium for both transformations, delivering full conversions and 93% isolated yield for the symmetrical etherification (affording **93**) and 88% for the cross‐etherification (to give unsymmetrical ethers **94**), out‐performing DMC, DCM and MeCN in both cases.

During the study, the authors identified a critical limitation with using DMC as the solvent that PC does not share. When benzyl alcohols bearing electron‐withdrawing groups (Cl, F, Br) are subjected to the reactions in DMC, the desired ether is not formed; instead, the DMC acts as a methyl carbonate‐transfer reagent to produce the corresponding methyl carbonates (53%–85% yield) as the main products instead of the desired ethers. This competing pathway arises because electron‐withdrawing groups destabilise the benzylic carbocation intermediate that is required for the normal etherification mechanism, as confirmed by the authors’ proposed catalytic cycle, making the carbocation pathway kinetically disfavoured. In the absence of a viable carbocation route, benzyl alcohol is instead diverted toward a transesterification pathway with DMC under Lewis acid catalysis. PC does not present this problem because, as a cyclic carbonate, it is inherently more resistant to acting as a carbonate‐transfer agent under these conditions. Beyond the reaction selectivity, the high polarity of PC promotes the ionic mechanism for both reactions, stabilising the benzylic carbocation intermediate and facilitating catalyst dissolution.

This paper provides a more complete description of product recovery from PC. The immiscibility of PC with petroleum ether allows selective product partition into the non‐polar phase, and the target compounds can be isolated through simple extraction or centrifugation, whilst the iron catalyst and ligand remain dissolved in the PC phase. This selective partitioning enables the direct recycling of both the solvent and the catalytic system, significantly enhancing the process efficiency. The solvent can be recovered by reduced‐pressure distillation, yielding PC at up to 80% purity for further use. Furthermore, the protocol exhibits an exceptionally high atom economy, as water is the only by‐product of both reactions, which reinforces the sustainability of this methodology.

**SCHEME 20:**
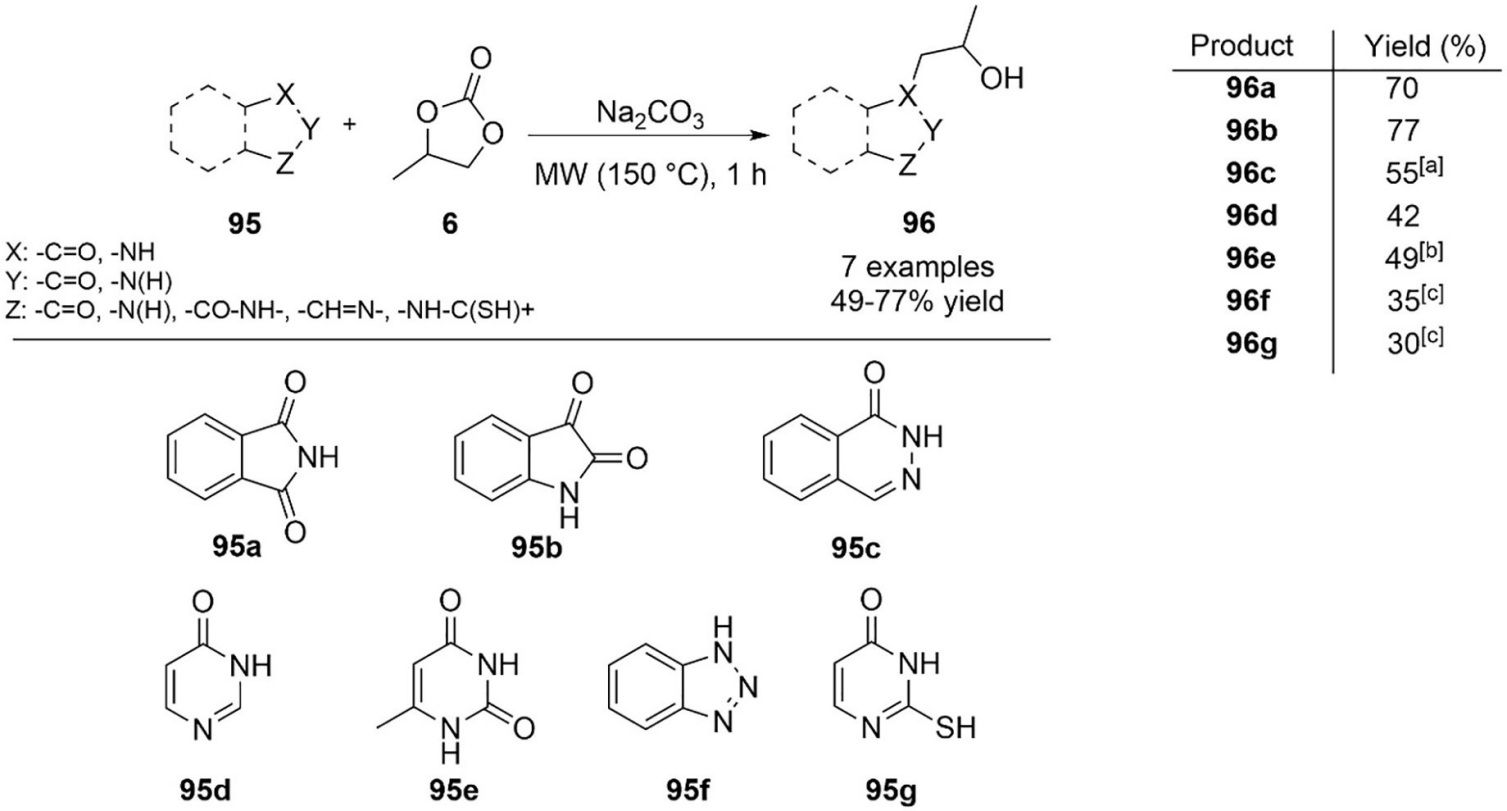
Use of PC as an alkylating agent. Reaction times: ^[a]^2 h, ^[b]^6 h and ^[c]^4 h.

Czompa and co‐workers explored the use of PC for the first time as both a solvent and a reagent for the *N*‐alkylation of *N‐*heterocycles, a transformation that is conventionally carried out with genotoxic alkyl halides (Scheme 20) [97]. The mechanistic basis for this transformation is the inherent electrophilic nature of the methylene carbon of the PC ring, which means it is susceptible to attack from a deprotonated nitrogen atom, thereby opening the ring and installing a 2‐hydroxypropyl group on nitrogen whilst releasing only CO_2_ as a by‐product. The authors exploited this reactivity for the synthesis of seven pharmaceutically relevant *N‐*heterocycles (**96**) under microwave irradiation in a single step using excess PC and solid Na_2_CO_3_ as the base. The authors demonstrated that the most important variable was the purity of PC, as with standard 99% pure PC, the ~1% water contaminant in the solvent directly competes with the nitrogen nucleophile, meaning that the water opens the cyclic carbonate in preference to the *N*‐heterocyclic substrate, thereby suppressing desired product formation. However, the use of anhydrous PC (99.7%) dramatically increased the yield without requiring the addition of any formal drying agent; for example, with phthalimide **95a,** the reaction went from no product formation to a 70% isolated yield. Under optimised reaction conditions (MW at 150 °C), product yields ranged from 55% to 77% across all the chosen substrates (**95a‐g**) with suitably acidic N─H bonds. Microwave heating was found to be consistently superior to conventional oil bath heating, as observed with phthalazine‐1(2*H*)‐one **95c** (50% under MW at 150 °C versus 28% in an oil bath at 170 °C). This is attributed to the high dipole moment and dielectric constant of PC, which make it an excellent microwave absorber, delivering fast and homogeneous heating throughout the reaction mixture.

**SCHEME 21:**
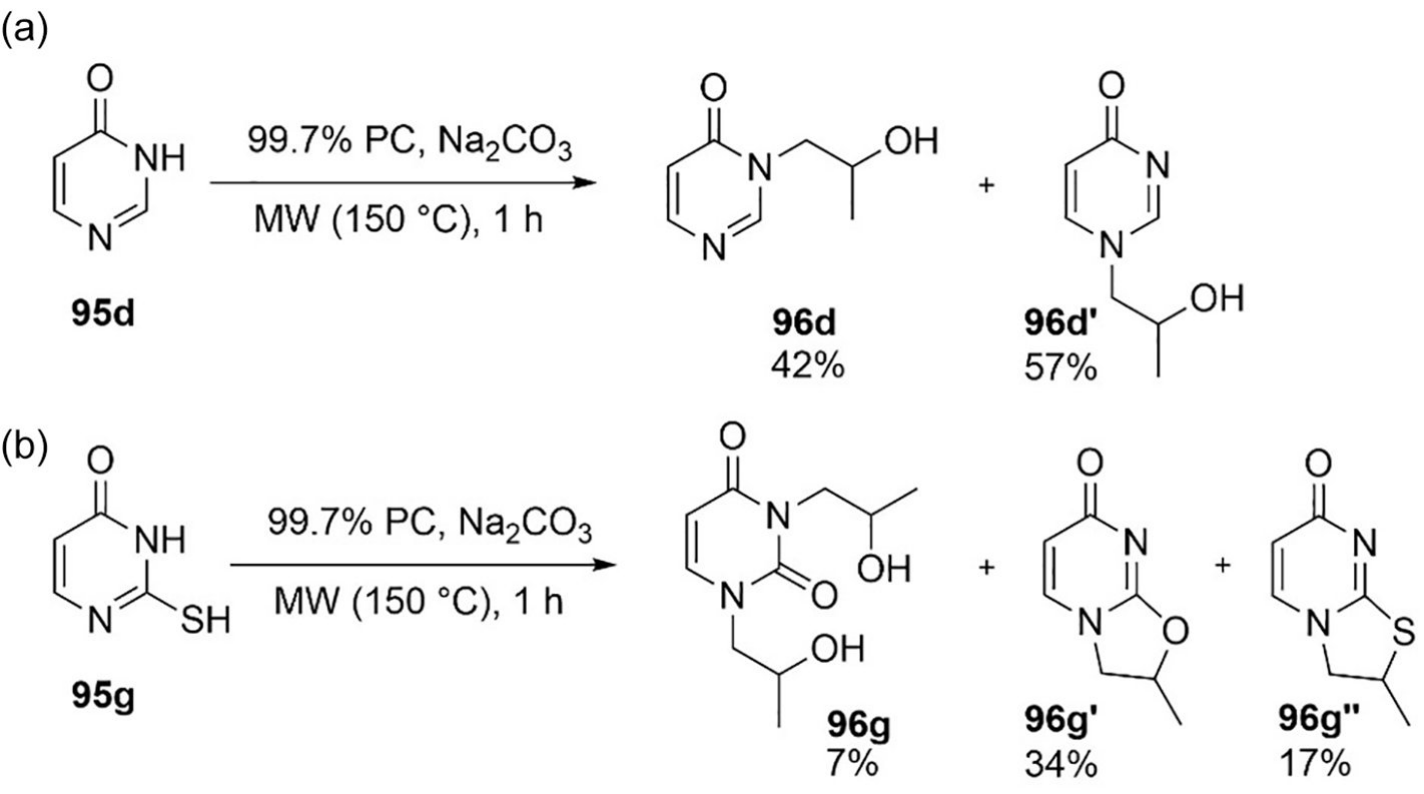
*N*‐Alkylation of pyrimidin‐4(3*H*)‐one **95d** and 2‐thiouracil **95g** and its by‐products.

Considering selectivity, the authors found that when a substrate contained two non‐equivalent nitrogen atoms, alkylation by PC occurred preferentially on the more acidic site. This was confirmed by DFT‐calculated p*K*_a_ values and was consistent across all substrates investigated. In pyrimidin‐4(3*H*)‐one **95d**, for example, alkylation at the more acidic position (p*K*_a_ 7.91) afforded the corresponding *N*‐alkylated product **96d** in 42% yield, whilst the less acidic nitrogen (N‐3, p*K*_a_ 8.40) led to **96d’** in 57% yield (Scheme 21a). An unexpected result occurred with 2‐thiouracil **95g**, as the initially formed monoalkylated product was found to undergo spontaneous intramolecular cyclisation, producing novel oxazole‐ (**96g’**) and thiazole‐fused (**96g’’**) ring systems in 34% and 17% yields, respectively, in addition to the bis‐alkylated product **96g** (Scheme 21b). The authors propose that the cyclisation is facilitated by the high temperature and anhydrous environment enabled by PC.

Although this methodology represents a new and mechanistically interesting approach for the synthesis of *N‐*heterocycles and that PC offers operational advantages (efficient and homogeneous microwave‐assisted heating), its role as a dual solvent‐reagent poses a sustainability challenge, as it requires an excess of PC (9–12 equivalents), resulting in poor atom economy. Furthermore, the current workup protocol involves a multi‐stage sequence: the reaction mixture is neutralised with aqueous HCl and extracted using traditional organic solvents (either ethyl acetate or chloroform), then washed with CuSO_4_ to remove the propylene glycol by‐product from PC ring opening, dried, evaporated, and lyophilised overnight, followed by final purification by column chromatography. Transitioning this protocol toward a simpler process could be a significant opportunity to enhance the overall sustainability. Specifically, replacing chloroform with a greener extraction solvent across all substrates would be a straightforward step, and a discussion of the recovery and recyclability of unreacted PC could also enhance its green profile, as no E‐factor or process mass efficiency assessment is reported to quantify a comparison against conventional alkyl halide routes.

**FIGURE 8:**
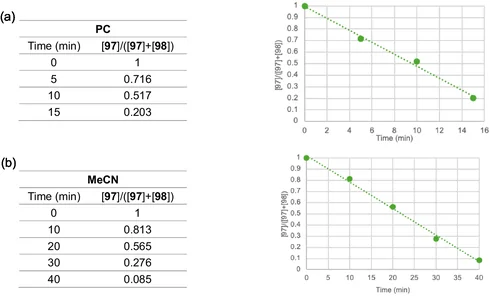
Rate of aryne formation in (a) PC and (b) MeCN. Aliquots taken from an aryne Diels–Alder reaction in PC and MeCN and a portion of precursor (**97**) versus the Diels–Alder adduct **98**, calculated from ^1^H NMR spectra.

**SCHEME 22:**
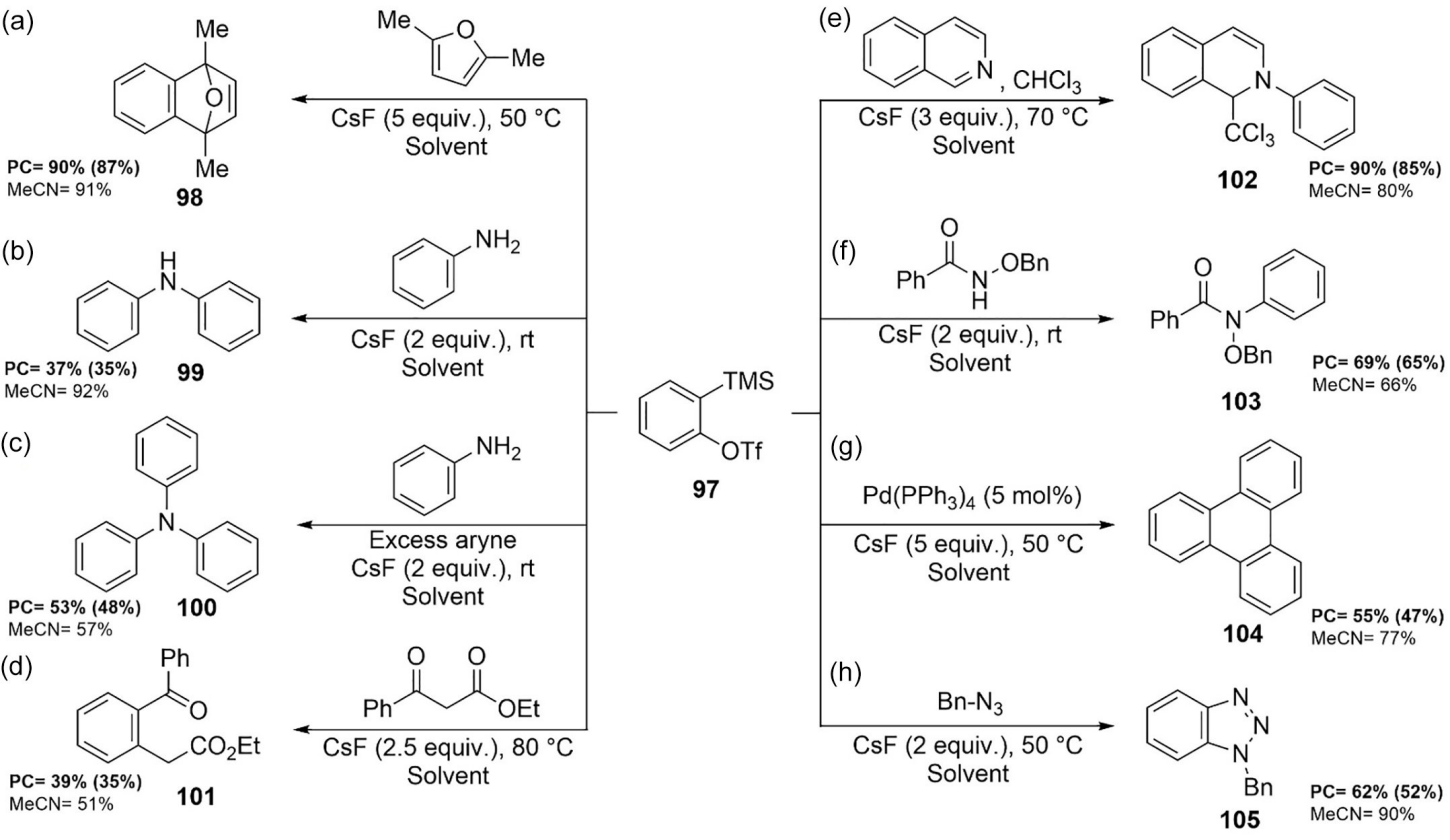
Common aryne transformation in PC and MeCN as solvents.

In 2025, Jones and co‐workers reported the first application of PC as a sustainable solvent in aryne chemistry, specifically in reactions involving *o*‐silylaryl triflate (*o*SAT) precursors, a class of reactions that traditionally relies on MeCN or THF [98]. PC was chosen due to its similar physicochemical properties to MeCN, suggesting that it could function as a direct replacement whilst offering a significantly better toxicity and sustainability profile. To ensure rigorous comparison, the performance of PC was benchmarked against MeCN using established literature conditions without further optimisation. In the benchmark Diels‐Alder cycloaddition, PC and MeCN delivered nearly identical NMR yields (90% and 91%, respectively), confirming PC as a fully competent alternative. Notably, a quantitative kinetic study revealed that the reaction rate in PC was twice as fast as in MeCN (Figure 8), an acceleration attributed to the higher dielectric constant (65 for PC vs. 36 for MeCN), which enhances the solubility and dissociation of the CsF salt. By more effectively activating the inorganic fluoride source responsible for generating the aryne intermediate, PC increases the overall concentration of the reactive intermediate and effectively drives the reaction forward through superior solvating power. The substrate scope for the Diels‐Alder reaction proved broad, with PC performing consistently alongside MeCN across electron‐rich, electron‐poor, and sterically hindered precursors, where yields typically fell within 5% of MeCN values. The suitability of PC for a wide range of mechanistically different aryne transformations in the literature was established by extending the study to formal aryne σ‐bond insertions, nucleophilic additions, multicomponent reactions and Pd‐catalysed cyclotrimerization (Scheme 22).

Interestingly, the study also identified a critical trade‐off in the selectivity observed in the mono‐arylation of aniline. PC yielded only 37% of the desired diphenylamine **99**, compared to 92% in MeCN, which was attributed to the rapid generation of arynes in PC, which led to deleterious over‐arylation (Scheme 22b,c). This finding was particularly instructive, as the same kinetic advantage that facilitated the Diels‐Alder cycloaddition can become more problematic in reactions requiring controlled, slower release of the aryne intermediate to prevent the formation of di‐arylated by‐products.

A current practical limitation discussed throughout this review is the high boiling point of PC, which complicates its removal via standard laboratory evaporation and typically requires additional steps for product isolation. In this study, the authors extracted the desired products with a small volume of hexane, which, whilst introducing an additional extraction step and a small amount of organic solvent to the process, is reasoned to still significantly reduce the total volume of organic solvent needed for the work‐up compared to conventional MeCN‐based reactions. Nevertheless, as in the previous study, the superior performance of PC could be more strongly supported with an E‐factor or mass efficiency analysis. Overall, the data presented in the study demonstrate that PC is a viable and broadly applicable green replacement for MeCN in aryne chemistry, with the added benefit of faster reaction rates in most cases.

### 2.8 Biomass Conversion

Yu et al. examined PC and γ‐valerolactone (GVL) as green co‐solvents in 1:1 (v/v) mixtures with water for the Sn(IV)‐catalysed conversion of bread waste to hydroxymethylfurfural (HMF, **106**) under microwave heating at 120 °C, a reaction that requires three sequential steps: starch hydrolysis, glucose isomerisation and fructose dehydration [99]. The choice of PC was motivated by its polar aprotic character and polarity comparable to DMSO (PC π* = 0.83, DMSO π* = 0.94), which identified it as a candidate to replace conventional but problematic solvents such as DMSO, DMA, and ionic liquids. This is noteworthy as PC is used in an aqueous binary system rather than as a neat solvent.

**FIGURE 9:**
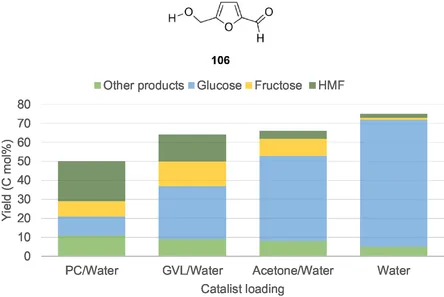
Product yields from the catalytic conversions of bread waste in different solvents under SnCl_4_ 5.5 mM loading, 5 wt/v% substrate in solvent mixture (1:1 v/v) or water at 120 °C for 10 min. Yield = product_Cmol_/substrate_Cmol_ *100%.

PC/H_2_O was the best‐performing system among the media tested (PC/H_2_O, GVL/H_2_O, PC/H_2_O, acetone/H_2_O and water), as observed in Figure 9. The key aspect to rationalise the superior performance of PC and GVL over acetone and water is not related to the substrate solvating ability but to their effect on the catalyst. In acetone/H_2_O and water, SnCl_4_ underwent hydrolysis upon heating, precipitating as inactive colloidal SnO_2_, confirmed by X‐ray diffraction analysis (HDR). This eliminated the Lewis acidic sites responsible for glucose‐to‐fructose isomerisation, making this the rate‐limiting step (47–59 mol% glucose remained unconverted). In PC/H_2_O and GVL/H_2_O, no SnO_2_ precipitation occurred, and the solution remained clear, indicating that both co‐solvents effectively suppressed metal hydrolysis and preserved the active Sn^4+^ species, achieving 20 mol% of HMF and leaving only ~9 mol% of glucose unconverted.

**FIGURE 10:**
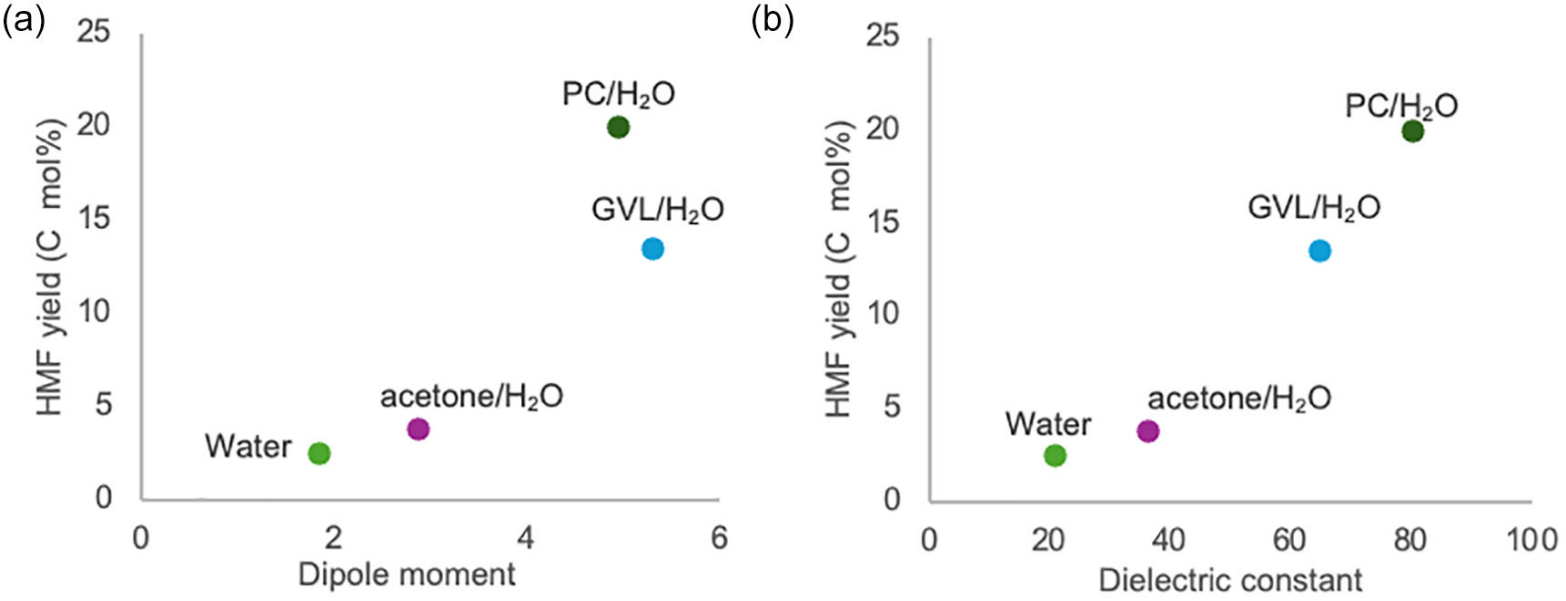
Correlation between HMF yields from bread waste conversions and (a) dipole moment of the organic co‐solvent and (b) dielectric constant of the organic co‐solvent. Conditions: 5 wt/v substrate in solvent mixture (1:1 v/v) or water at 120 °C for 10 min. Yield = product_Cmol_/substrate_Cmol_ * 100%.

The authors propose that the good performance of PC and GVL is due to the preferential coordination to Sn^4+^ of organic solvent molecules, thereby reducing metal‐water interactions and preventing hydrolysis. A positive correlation was observed between the dipole moment of the co‐solvent and HMF yields. This supports the interpretation that the higher the dipole moment, the stronger the ligand‐like interaction with the metal centre. PC has a dipole moment of ~4.9 days, higher than that of GVL (~4.2 days) and considerably more than that of acetone (~2.9 days), which follows the observed performance ranking. A similar link was found with the dielectric constant, which also governs microwave absorption efficiency: PC (*ε* = 64) heats more effectively under MW irradiation than acetone (*ε* = 20.7) or GVL (*ε* = 36.4), further contributing to its performance advantage (Figure 10).

**FIGURE 11:**
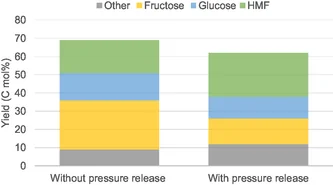
Product yields resulted from the catalytic conversion of bread waste in PC/H_2_O with and without pressure release. Conditions: SnCl_4_ 5.5 mM loading, 5 wt/v% substrate in solvent mixture (1:1 v/v) or water at 120 °C for 2.5 min each for the first and second heating. Yield = product_Cmol_/substrate_Cmol _* 100%.

Interestingly, the superior reactivity of the PC/H_2_O solvent mixture over GVL/H_2_O is not explained solely by these solvent properties. The authors identify a second, distinct mechanism, where PC partially decomposes in hot water, releasing CO_2_ and generating autogenous in‐vessel pressure, approximately 10 bar at the optimal 7.5 min reaction time, rising to 30 bar at 40 min. On the other hand, GVL/H_2_O only developed ~4 bar under the same conditions. A pressure‐release experiment directly confirmed this effect: releasing the pressure midway through the reaction reduced the HFM yield by 25% compared to the sealed control (Figure 11). The authors proposed that the elevated CO_2_ pressure enhances both Lewis and Brønsted acid catalysis, possibly by forming carbonic acid as an additional proton source or by altering the solvation environment of the active species, though it was acknowledged that this mechanism is not yet fully understood. This is a novel finding within the context of PC used as a solvent, as it is the first time that this tendency to decompose in hot water has been observed. Moreover, instead of being a drawback, the in situ pressure generated is viewed as an asset that accelerates the reaction. It is worth noting that, at the optimal reaction time, PC decomposition remained below 5% v/v, indicating that the bulk of PC is preserved and that this pressure effect is achievable without significant solvent consumption.

Whilst a direct experimental comparison was not performed in this study, the performance of PC is highly competitive with established literature benchmarks, where the system achieved a maximum yield of ~20 mol% at 120 °C in only 7.5 min, representing a significant optimisation over 140 °C in 40–60 min typically required for DMSO and MeCN [100].

A key finding in this study is the partial decomposition of PC in hot water. Whilst this decomposition helps accelerate the reaction, it is a double‐edged sword as the same conditions that speed up the desired conversion also promote side reactions like rehydration and polymerisation. This creates a very narrow selectivity window, meaning that the reaction must be stopped precisely at the maximum yield to avoid rapid product degradation. Furthermore, because this kinetic advantage is linked to the solvent’s own decomposition, it raises important questions about solvent consumption and the feasibility of recovering PC in larger‐scale applications.

### 2.9 Biocatalysis

**SCHEME 23:**
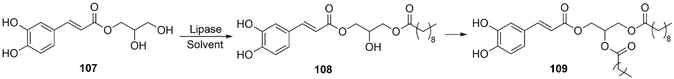
Acylation of glyceryl‐1‐caffeate (GC) **107** to GC‐3‐decanoate **108** and GC‐2,3‐didecanoate **109**.

In 2021, Cumming and Marshall extended the application of PC into biocatalysis, investigating its compatibility with enzymatic reactions, specifically lipase‐catalysed esterification (Scheme 23) [101]. This is a significant expansion, as lipases are among the most industrially relevant enzymes in food, pharmaceutical and cosmetic chemistry, with MeCN, 2‐methyl‐2‐butanol (2M2B) and acetone typically used in these reactions [102]. In this study, PC was used as the solvent for lipase‐catalysed esterification of glycerol‐1‐caffeate (GC) **107** with decanoic and palmitic acids (*Thermomyces lanuginosus lipase* TLIM and Novozym 45). PC was selected for its polarity and hydrogen bond acceptor properties, which are similar to those of MeCN and compatible with lipase activity. Furthermore, in this instance, the low volatility of PC offers a unique advantage by allowing the reaction to be performed under vacuum. This enables the continuous removal of water, without the need for molecular sieves, which is a distinct operational advantage compared to conventional solvents.

**FIGURE 12:**
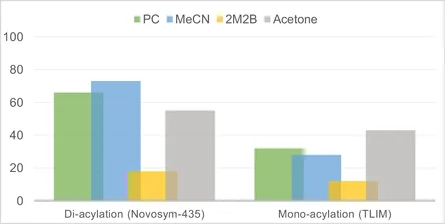
Effect of solvent and immobilised lipase on the conversion of GC‐3‐decanoate **108** and GC‐2,3‐didecanoate **109**. *Note:* all reactions optimised: 55 °C, 24 h, 5 equiv., decanoic acid, closed system.

In initial unoptimised trials, PC performed comparably with traditional solvents: 66% in PC versus 73% in MeCN for the di‐acylated product **109** with Novozym‐435 and 32% in PC versus 43% in 2M2B for the mono‐acylated product **108** with TLIM (Figure 12). However, once conditions were optimised for PC, conversions improved significantly, reaching 79% for di‐acylated product **109** (Novozym‐435 at 80 °C) and 77% for mono‐acylated product **108** (TLIM at 50–60 °C). Importantly, the enzymes maintained their natural regioselectivity regardless of the solvent, confirming that PC does not interfere with the enzyme’s active site or substrate recognition.

A significant aspect of this study is the reusability of both the immobilised lipase and PC. After each cycle, products were extracted with diisopropyl ether (DIPE), taking advantage of its immiscibility with PC and allowing the PC/lipase system to be recharged with a fresh substrate for four consecutive cycles with minimal loss of activity. However, this recovery strategy revealed a critical limitation: the efficiency of DIPE extraction depends heavily on the lipophilicity of the product. For example, highly lipophilic compounds were extracted with 93% efficiency, requiring only two washes; however, less lipophilic products proved much more difficult to isolate, with only 11% efficiency after two washes and 27 extractions required to recover 95% of the product. This means that whilst PC reuse is practical for hydrophobic products (i.e. di‐acylated **109**), the work‐up for mono‐acylated compounds (**108**) remains solvent‐intensive and impractical for large‐scale use. Furthermore, employing DIPE introduces safety concerns, as it can form explosive peroxides, partially offsetting the green benefits of using PC. The authors acknowledge the possibility of using an alternative work‐up with a PC miscible solvent (such as methyl‐*tert*‐butyl ether, MTBE, or diethyl ether) and washing with water to eliminate PC; however, they recognise this could complicate PC reuse and thus eliminate the most prominent advantage of the reaction protocol.

A pattern of processing limitations recurs across multiple studies in this review.

Overall, this study confirms that PC is a viable green alternative for lipase‐catalysed esterification: its ability to facilitate vacuum‐driven water removal is a meaningful practical advantage. Whilst the product‐dependent extraction efficiency limits its general application for all compound types and is another example of the processing limitations of PC covered across the review, the study does provide a comprehensive framework for evaluating reaction performance, enzyme stability and solvent recovery in green biocatalysis.

### 2.10 Degradation Reactions

**SCHEME 24:**
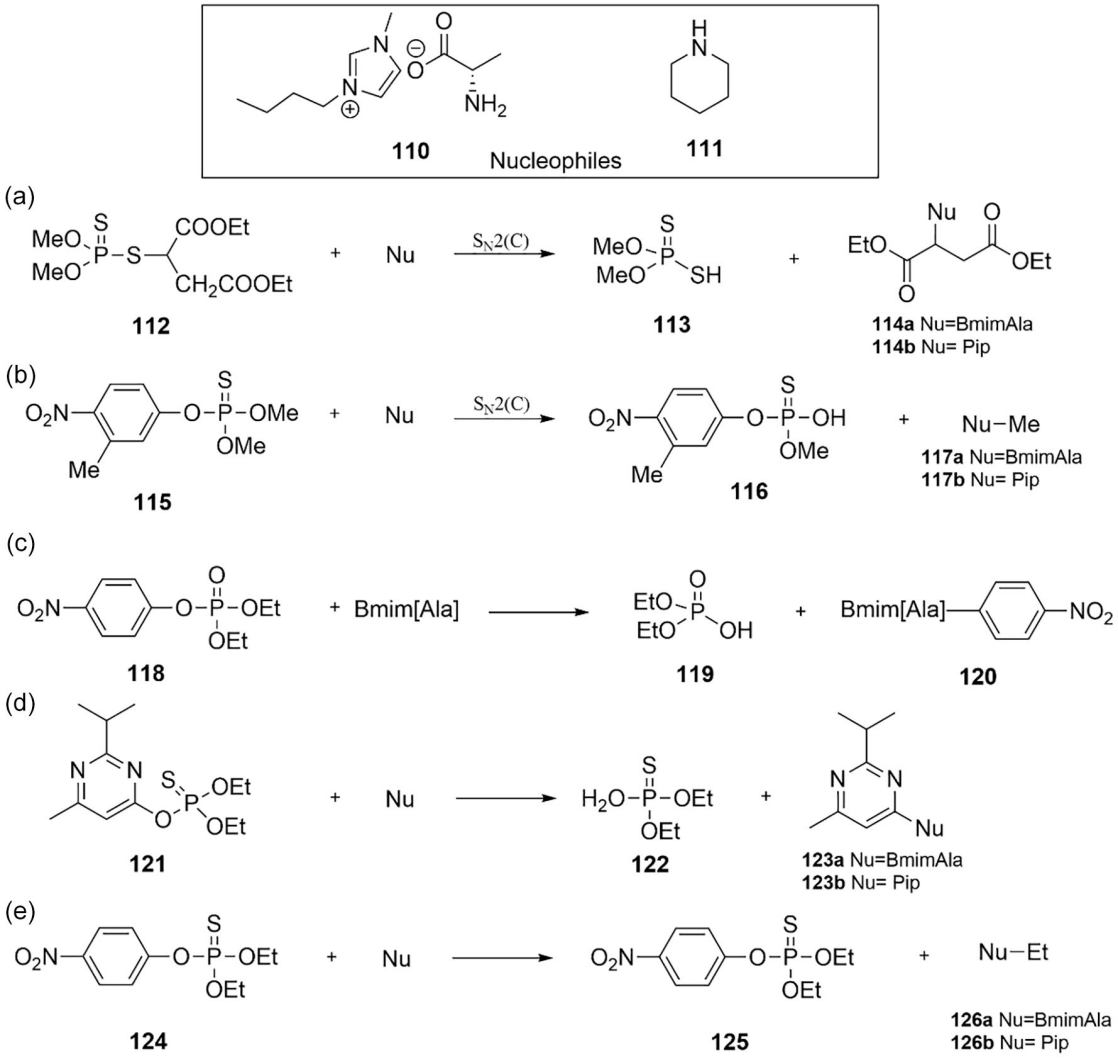
Reaction pathway for the degradation of organophosphate pesticides **112**, **115**, **118**, **121** and **124** by both nucleophiles (Bmim[Ala] **110** and piperidine **111**) in PC at room temperature.

Organophosphorus pesticides (OPPs) are a major global environmental concern due to their accumulation in soil and groundwater. Research focuses on their chemical degradation by breaking down toxic triesters into safer, less lipophilic phosphates via nucleophilic attack. Millán et al. investigated the use of PC as a solvent for this type of process, at room temperature and under microwave heating, testing five OPPs (malathion **112**, fenitrothion **115**, paraoxon **118**, diazinon **121** and parathion **124**) against two nucleophiles: the ionic liquid Bmim[Ala] **110** and piperidine **111** (Scheme 24) [103].

PC proved to be a highly enabling medium as it actively controlled the selectivity of the degradation mechanism. For example, all aromatic pesticides reacted exclusively through the S_*N*_2(C) pathway (i.e. attack on the aliphatic carbon see Scheme 24a,b), producing a single clean degradation product, whereas the same reactions carried out in ionic liquids and other bio‐based solvents produced mixtures from three competing reaction pathways. The selectivity is attributed to the low basicity of PC, which favours soft nucleophiles attacking the soft aliphatic carbon of OPP in comparison to the phosphorous centre. This translates directly into cleaner degradation profiles, which is a particularly valuable practical benefit, as a single clean product is much easier to handle than a complex mixture.

In terms of reaction rate (Table 16), PC also outperformed other green solvents: for fenitrothion (**115**) degradation with piperidine at room temperature, PC/piperidine gave *t*_1/2_ = 23 versus 13.2 min in Bmim[*NTf*_2_]/piperidine and 210 min in ethyl lactate/piperidine, a marked difference that positions PC as the best‐performing green solvent tested in this reaction class. For malathion **112**, both nucleophiles afforded near‐identical half‐lives (~10 min) in PC at room temperature, confirming that even bulkier, less classical nucleophiles such as Bmim[Ala] perform on a par with piperidine when the substrate is inherently reactive. The combination of PC with microwave heating further extended the scope; both diazinon **121** and parathion **124** showed no reactivity with either nucleophile at room temperature but reached 90%–100% conversions in 30 min under MW heating at 90–110 °C in PC (note: conventional oil bath heating at the same temperatures gave only modest conversions). This is consistent with the efficient MW absorption that PC’s high dielectric constant and dipole moment provide.

**TABLE 16:** Half‐lives (*t*_1/2_) for the degradation of pesticides (**112**, **115**, **118**, **121 **and **124**) in PC at room temperature and MW irradiation.^a^

	Room temperature (*t*_1/2_), min		MW – Temp. for complete conversion	
	Bmim[Ala], 110	Piperidine, 111	Bmim[Ala], 110	Piperidine, 111
Malathion, **112**	10.7 ± 0.60	9.9 ± 0.6	Not needed	Not needed
Fenitrothion, **115**	425 ± 11.4	23.1 ± 0.6	Not needed	Not needed
Paraoxon, **118**	1203 ± 96.1	—	120 °C	Not tested
Diazinon, **121**	—	—	100 °C	100 °C
Parathion, **124**	—	—	90 °C	110 °C

a All reactions in PC as a solvent. MW conditions: 30 min irradiation, conversions >90%; —, no reaction observed.

However, the results in this study also reveal important limitations, as not all pesticide‐nucleophile combinations benefit equally from using PC: fenitrothion **115** degraded with *t*_1/2_ = 23 min using piperidine but *t*_1/2_ = 425 min using the nucleophile Bmim[Ala], showing that PC cannot compensate for a poorly matched nucleophile‐substrate pair. Similarly, paraoxon **118** showed no reactivity with piperidine at room temperature despite being fully degradable with Bmim[Ala], which illustrates that the choice of the nucleophile in PC can be the difference between a functional and a completely non‐functional system. Additionally, paraoxon required the highest MW temperature of all (120 °C) despite having a theoretically more electrophilic *P*═O centre than the *P*═S centres of parathion or diazinon, an anomaly that the authors acknowledge but do not fully resolve. This suggests that PC, the nucleophile, and the pesticide interact in a more complex manner than currently understood. Furthermore, the study reports only conversions measured by ^31^P NMR with no product isolation, no PC recovery protocol and no comparison against aqueous degradation methods. This means that whilst PC clearly works well at the reaction level, its viability as part of a complete, scalable decontamination process has not been demonstrated.

## 3 Outlook and Summary

Building on the previous review of the topic by Jones Junior, Da Silva and co‐workers in 2016 [7], the body of recent literature reviewed here collectively establishes propylene carbonate as one of the most versatile and broadly validated green solvents currently available for organic synthesis, with applications covering transition‐metal‐catalysed cross‐coupling and carbonylation reactions, oxidative transformations, asymmetric hydrogenation, electrosynthesis, CO_2_‐based chemistry, peptide synthesis, biocatalysis, biomass conversion and degradation reactions. Unlike many green solvents, where utility has been demonstrated within a narrow reaction class, PC has now been validated across a wide variety of chemical transformations, with a consistent physicochemical rationale to explain its observed performance.

The key to the effectiveness of PC is its exceptionally high dielectric constant (*ε* = 64), which is the highest among the green solvents discussed in this review (e.g. DMC, GVL and 2‐MeTHF). This value far exceeds that of common solvents like acetonitrile (*ε* = 37), acetone (*ε* = 21) and DMSO (*ε* = 46), whilst carrying none of the toxicological concerns. The high dielectric constant is one of the reasons that PC works so well across a wide range of reaction classes. For example, in Pd‐catalysed reactions, PC stabilises the charged transition states and ionic intermediates of the catalytic cycle, as confirmed by consistent trends across Suzuki–Miyaura, Heck, C─H activation, carbonylative Sonogashira, phenoxycarbonylation and aminocarbonylation studies. In electrosynthesis, the dielectric constant governs ionic conductivity and electrolyte dissociation in binary PC‐DMC systems, whereas in biomass conversion, the solvent prevents catalyst decomposition (SnCl_4_ into SnO_2_) by coordinating to the metal centre. In aryne chemistry with *o*‐silylaryl triflate precursors, PC enhances the dissociation of the inorganic fluoride sources and was found to double the rate of aryne generation relative to reactions conducted in MeCN. Beyond the dielectric constant, PC possesses a high dipole moment (~4.9 days) that facilitates absorption of microwave energy to deliver homogeneous volumetric heating that consistently out‐performs conventional oil bath heating at higher temperatures across *N‐*alkylation, pesticide degradation, biomass conversion and carbonylative reactions. This property also translates into shorter reaction times, lower energy consumption and often improved yields.

It must be acknowledged, however, that PC's relatively high viscosity (*η* = 2.53 mPa s) represents a physical property that can work against its advantages in certain contexts. As discussed in Sections 2.2 and 2.3 (oxidation and asymmetric hydrogenation reactions), high viscosity limits the diffusion of reactive species such as TEMPO in electrochemical oxidations and impairs hydrogen solubility and mass transfer in asymmetric hydrogenation reactions, in the latter case requiring higher H_2_ pressures and longer reaction times relative to less viscous solvents such as DCM. The viscosity of PC, therefore, represents a practical constraint that must be considered when evaluating its suitability for a given transformation, and the use of binary solvent systems, such as PC–DMC mixtures, has been shown to offer an effective means of tuning viscosity without sacrificing the conductivity and solvation benefits that PC provides.

Beyond these established physicochemical properties, a series of novel discoveries about the behaviour of PC as a solvent emerged across this review. In iron‐catalysed etherification, the cyclic structure of PC was shown to prevent a competitive transesterification process, unlike linear carbonate solvents such as DMC, which were found to act as methyl carbonate transfer agents under certain etherification conditions. In phosphomolybdic acid‐catalysed carbohydroxylation, ESI‐MS analysis provided the first direct spectroscopic evidence of PC binding to a catalyst, serving as an active co‐participant in the catalytic cycle rather than as a passive reaction medium. In Sn(IV) catalysed biomass conversion, the partial decomposition of PC in hot aqueous media was demonstrated to generate autogenous CO_2_ pressure (~10 bar at optimal conditions) that accelerates the reaction, representing a previously unrecognised mechanism specific to PC in aqueous systems. In pesticide degradation, PC enforced an exclusive reaction via an S_*N*_2(C) mechanism across all aromatic substrates, which was not replicated in ionic liquids or other bio‐based solvents. This was attributed to the lower basicity of PC, favouring soft nucleophile attack at the aliphatic carbon over the harder phosphorous centre. In asymmetric hydrogenation, PC did not interfere with the sensitive chiral metal‐ligand environments and was therefore able to reproduce the enantioselectivities achieved in traditional solvents like DCM and THF. Furthermore, it was shown that the low volatility of PC enables vacuum‐driven continuous water removal in lipase‐catalysed esterification without the need for molecular sieves, which offered a key operational advantage over conventional volatile solvents. Importantly, full enzyme regioselectivity is preserved in this biocatalytic process, confirming that PC does not disrupt lipase active site geometry.

An important finding that emerges across several of the studies is that PC is not always merely a spectator solvent in all transformations. Under certain conditions, notably in the presence of strong bases, strong acids or reactive nucleophiles, PC can itself act as a reagent, a behaviour that carries both significant opportunities and important limitations. On the opportunity side, the susceptibility of the cyclic carbonate ring to nucleophilic attack under basic conditions has been deliberately exploited in the microwave‐assisted *N*‐alkylation of *N*‐heterocycles, where PC acts simultaneously as a solvent and alkylating agent, installing a 2‐hydroxypropyl group on nitrogen whilst releasing only CO_2_ as a waste by‐product. This dual solvent‐reagent functionality eliminates the need for genotoxic alkyl halides and represents a genuinely novel synthetic application of PC. Similarly, the ring opening of PC under basic aqueous conditions was observed in the Suzuki–Miyaura reaction with substrates bearing acidic N─H groups, where the resulting 2‐hydroxypropyl adduct was formed as a novel compound, highlighting the potential of PC's reactivity to generate new chemicals under green conditions. However, on the limitation side, this same reactivity imposes important constraints on the scope of reactions for which PC can be considered a suitable solvent. As discussed across Sections 2.1, 2.2 and 2.6 (Pd‐catalysed, oxidation, addition reactions and C─C/C─O bond formation reactions), PC must be used with caution in the presence of strongly basic or nucleophilic reaction conditions, strongly acidic conditions, where hydrolysis of the cyclic carbonate can occur with release of CO_2_, and substrates or intermediates that are themselves susceptible to ring opening. In such cases, the solvent is no longer chemically unreactive, and its participation in the reaction must be carefully considered as part of the reaction design, as these findings underscore the importance of evaluating not only the polarity and thermal properties of PC but also its chemical stability under specific conditions of each intended application.

In general, the performance of PC varies depending on the reaction; it is usually a strong competitor for traditional polar aprotic solvents. In transformations where polarity is the determining factor—the majority of ionic and transition‐metal‐catalysed transformations—PC consistently matches or out‐performs DMF, MeCN, and DMSO both in yield and selectivity. This allows the traditional organic solvent to be replaced with a solvent that is less hazardous and biodegradable, possesses negligible vapour pressure and has a toxicity profile (LD_50_ > 29 g/kg in rats) that makes it suitable for pharmaceutical and food‐related applications. In carbonylative Pd/C reactions that required a phosphine ligand in DMF, PC allowed the process to work well under ligand‐free conditions, suggesting that the more polar environment can partially compensate for the absence of a stabilising co‐ligand at the catalyst surface. For radical‐initiated dioxygenation, the non‐nucleophilic nature of PC was the defining property that directed selectivity towards bisperoxide products, whilst water preferred the formation of the corresponding diol products; this is a level of control that traditional solvents do not offer. Similarly, PC outperformed both DMF and DCM as a solvent in peptide synthesis, achieving a 93% yield compared to 81% and 83%, respectively, whilst also producing a more favourable impurity profile in the solid‐phase synthesis of bradykinin. However, it must be noted that PC is not universally superior; in CO_2_ cycloaddition to afford limonene oxide, DMF outperformed PC due to the amide functional group acting as a nucleophilic activator of CO_2_, a synergistic contribution that is not replicated by the less Lewis basic carbonate group in PC. Elsewhere, in allylic oxidation of *cis*‐Jasmone, the high polarity of PC promoted substrate isomerisation over the desired oxidation, whilst the observed increased rate of aryne generation in PC caused undesired over‐arylation of aniline. As such, whilst the high polarity of PC is usually a positive aspect, it presents a limitation in certain cases, as it can interfere with reactions involving substrates prone to isomerisation, processes that require a slow release of intermediates, or target compounds that are themselves sensitive to highly polar environments.

The most persistent limitation of PC discussed within this review is product recovery, as the high boiling point (243 °C) makes solvent removal by evaporation extremely challenging using standard laboratory apparatus. Approaches to tackle this issue include vacuum distillation, liquid–liquid extraction with a non‐miscible solvent (which relies on the lipophilicity of the product), or absorption‐based separation at the work‐up stage. Importantly, most of the studies reviewed here do not report the recovery of PC and do not address to what extent the green profile of the transformation in PC is offset by the energy‐ or solvent‐intensive work‐up procedures employed. Perhaps the best exception to this observation was presented in the iron‐catalysed etherification work from Litim and co‐workers, where the immiscibility of PC with petroleum ether enabled simultaneous product isolation and catalyst/solvent recovery, demonstrating that sustainable PC use is achievable when the relative physical properties of the reaction mixture are considered during the work‐up design. Shafiee and co‐workers’ oxazolone synthesis represents an alternative positive example, where product precipitation upon water addition allowed recovery by simple filtration and the reuse of PC over five cycles with only a small loss in yield. This demonstrates that when reaction products are sufficiently insoluble in PC, the high boiling point becomes an asset rather than a limitation, as the solvent can be recovered without any additional steps.

To consolidate these observations, Table 17 summarises the principal advantages and limitations of propylene carbonate, bringing together its physicochemical properties, reaction‐specific performance and operational behaviour. This comparative overview highlights how PC’s good polarity, thermal stability, broad functional group compatibility and favourable environmental profile support many of its successes, whilst viscosity, chemical reactivity under specific conditions and challenges in solvent recovery set the limits of its applicability. By presenting these aspects side by side, the table provides a practical framework for evaluating when PC is likely to enhance a transformation and when its intrinsic properties may create constraints that must be considered during reaction design.

**TABLE 17:** Summary of the advantages, benefits, disadvantages and limitations of PC as a green alternative in organic synthesis.

	Advantages/benefits	Disadvantages/limitations
Polarity and dielectric properties	High dielectric constant (*ε* = 64) stabilises ionic intermediates, enhances Pd‐catalysed cycles, accelerates aryne formation and improves electrolyte dissociation	Excessive polarity can promote isomerisation or interfere with sensitive intermediates
Microwave absorption and heating	High dipole moment (~4.9 days) enables efficient MW absorption leading to faster reactions, lower energy use and better yields	Rapid heating can increase side reactions or over‐activation in some systems
Chemical reactivity	Can act as solvent‐reagent (e.g. green *N*‐alkylations), enabling novel ring‐opened products without hazardous alkylating agents	PC is not always inert; susceptible to ring opening under strongly acidic, basic or nucleophilic conditions, causes hydrolysis or reaction with substrates
Functional group compatibility	Broad compatibility across many reaction classes, often matching or outperforming DMF, MeCN and DMSO in yield and selectivity	In some transformations (e.g. CO_2_ cycloaddition, allylic oxidation), PC is outperformed by DMF or other solvents due to lower Lewis basicity or excessive polarity
Environmental and safety profile	Biodegradable, non‐corrosive, low toxicity, non‐volatile; suitable for pharmaceutical/food applications; produced via atom‐economical CO_2_ valorisation	Its high boiling point (243 °C) complicates solvent removal, requiring energy‐intensive work‐up or alternative separation techniques; many studies do not report PC recovery, raising questions about its true sustainability profile
Viscosity and mass transfer	High viscosity can stabilise catalysts, and PC can be reused across multiple reaction cycles with minimal loss of efficiency due to thermal stability	High viscosity (*η* = 2.53 mPa s) reduces diffusion and hydrogen solubility, slowing mass transfer in hydrogenation and electrochemical oxidation reactions, requiring higher pressures or longer reaction times
Operational advantages	Immiscibility with non‐polar solvents can simplify product isolation and solvent recovery	Partial miscibility with water can complicate aqueous extractions; carbonyl band in IR spectrum can complicate reaction monitoring

Looking ahead, the evidence accumulated in this review points to several clear priorities, starting with the need to develop better and more sustainable work‐up protocols that can take advantage of the immiscibility of PC with non‐polar solvents, for extraction, and the high boiling point, for solvent recovery, as the absence of these methods remains the greatest gap between successful reactions in a green solvent and a truly sustainable overall process. Furthermore, the chemical versatility of PC, including its ability to coordinate with metal catalysts, generate pressure through controlled decomposition, exhibit direct reaction selectivity and interact with intermediates, could be embraced as a deliberate design principle in future research. Similarly, the consistent rate acceleration effect observed under microwave irradiation, across multiple reaction classes, could be integrated into the specific design of new synthetic methods rather than an incidental observation. Finally, the chemical stability of PC under strongly basic, acidic and nucleophilic conditions must be systematically mapped so that researchers can make informed decisions regarding appropriate solvent choice. Taken together, the work reviewed here establishes a compelling case for PC as a highly promising green solvent, whilst also highlighting gaps in sustainability commentary amongst current reports as well as identifying the steps necessary to transition from laboratory‐scale synthesis into large‐scale industrial processes.

Overall, the available literature supports PC as a highly versatile green alternative but not necessarily as a guarantee of overall process sustainability, emphasising the need to evaluate solvent choice within the broader context of reaction conditions and workup requirements.

## Funding

This work was supported by the Fundación Politécnico.

## Conflicts of Interest

The authors declare no conflicts of interest.

## Data Availability

Data sharing is not applicable to this article as no datasets were generated or analysed during the current study.
